# Bioinformatics Analysis of Allele Frequencies and Expression Patterns of ACE2, TMPRSS2 and FURIN in Different Populations and Susceptibility to SARS-CoV-2

**DOI:** 10.3390/genes12071041

**Published:** 2021-07-05

**Authors:** Mohammad Tarek, Hana Abdelzaher, Firas Kobeissy, Hassan A. N. El-Fawal, Mohammed M. Salama, Anwar Abdelnaser

**Affiliations:** 1 Bioinformatics Department, Armed Forces College of Medicine, Cairo 12622, Egypt; mohammadtareq459@gmail.com; 2 Institute of Global Health and Human Ecology, School of Science and Engineering, The American University in Cairo, Cairo 12622, Egypt; hana-abdelzaher@aucegypt.edu (H.A.); hassan.elfawal@aucegypt.edu (H.A.N.E.-F.); Mohamed-Salama@aucegypt.edu (M.M.S.); 3 Department of Emergency Medicine, McKnight Brain Institute, University of Florida, Gainesville, FL 32610, USA; firasko@gmail.com; 4 Department of Biochemistry and Molecular Genetics, American University of Beirut, Beirut 1107 2020, Lebanon

**Keywords:** COVID-19, SARS-CoV-2, ACE2, TMPRSS2, FURIN, variants, eQTLs

## Abstract

The virus responsible for the COVID-19 global health crisis, SARS-CoV-2, has been shown to utilize the ACE2 protein as an entry point to its target cells. The virus has been shown to rely on the actions of TMPRSS2 (a serine protease), as well as FURIN (a peptidase), for the critical priming of its spike protein. It has been postulated that variations in the sequence and expression of SARS-CoV-2’s receptor (ACE2) and the two priming proteases (TMPRSS2 and FURIN) may be critical in contributing to SARS-CoV-2 infectivity. This study aims to examine the different expression levels of FURIN in various tissues and age ranges in light of ACE2 and TMPRSS2 expression levels using the LungMAP database. Furthermore, we retrieved expression quantitative trait loci (eQTLs) of the three genes and their annotation. We analyzed the frequency of the retrieved variants in data from various populations and compared it to the Egyptian population. We highlight FURIN’s potential interplay with the immune response to SARS-CoV-2 and showcase a myriad of variants of the three genes that are differentially expressed across populations. Our findings provide insights into potential genetic factors that impact SARS-CoV-2 infectivity in different populations and shed light on the varying expression patterns of FURIN.

## 1. Introduction

In February 2020, the most recent addition to the coronavirus family (severe acute respiratory syndrome coronavirus-2 or SARS-CoV-2 in short) was already taking the world by storm. It was spreading at such a rapid rate that the WHO made the announcement that the virus and its associated pneumonia were in fact a pandemic public health menace [1]. As of February 2021, over 110 million people have contracted SARS-CoV-2 worldwide, 2.5 million of whom have died [2]. One of the most intriguing phenomena of this disease is the variation in different populations’ susceptibility to it, as well as the variations in its severity [3]. Egypt has been one of the countries showcasing lower infectivity rates than anticipated, and speculations as to why this is the case include underdeveloped surveillance systems, underrated reporting, temperature and different viral strains, among others [4]. With the current ambiguity surrounding trends in SARS-CoV-2 infectivity, there is a dire need for attempts to gain insights into the mechanisms governing this infection. Evidence suggests that this variation in population infectivity and severity may be accounted for by expression quantitative trait loci (eQTL) (genetic loci explaining part of the variations of gene expression phenotypes) and differences in their frequencies [5]. EQTLs are identified through genome-wide association studies (GWAS), which analyze associations between genetic variation markers and gene expression levels in large cohorts of individuals [6].

SARS-CoV-2 receptor recognition and binding are a deterministic factor in the success or failure of the process of viral infection. Similarly to SARS-CoV, SARS-CoV-2 utilizes the angiotensin-converting enzyme 2 (ACE2) as an entry point to its target cell [7]. ACE2 is widely expressed in a variety of organs in the human body, including the lungs, intestine, and testis [8]. Viral entry occurs through the high-affinity binding of the virus’s spike (S) protein to ACE2 [9]. However, the S protein requires priming, as only the surface unit, S1, of the S protein is capable of engaging ACE2. This priming process has been shown to occur mainly via the action of the cellular serine protease 2 (TMPRSS2) [10]. As such, TMPRSS2 activity is another deterministic factor in SARS-CoV-2’s colonization of host cells. DNA polymorphisms in both *ACE2* and *TMPRSS2* have been linked to variations in susceptibility to SARS-CoV-2 infection [11]. Other cellular proteases have been suggested to play a role in SARS-CoV-2 infectivity. The MERS-CoV virus was already proven to undergo a two-step priming process that started with FURIN S1/S2 cleavage of the S protein in infected cells, followed by TMPRSS2-mediated S20 cleavage [12]. This same FURIN-mediated cleavage has been demonstrated to occur in the SARS-CoV-2 S protein, which contains a multibasic FURIN cleavage site [13].

*ACE2* eQTLs, as well as *TMPRSS2* eQTLs and their frequencies, have been compared across populations and linked to variations in SARS-CoV2 susceptibility [14,15,16]. *FURIN* eQTLs have been suggested to play a role in SARS-CoV-2 infectivity and their frequency in Middle Eastern populations has been found to differ from European populations [17]. FURIN cleavage has been regarded as a crucial event in the cellular entry of SARS-CoV-2; however, few investigations have focused on the expression patterns of this protease, in comparison to investigations focused on the cellular receptor ACE2 and TMPRSS2 [18]. In this paper, we attempt to explain variations in SARS-CoV-2 infectivity by further exploring the differential expression of *FURIN* in light of *ACE2* and *TMPRSS2* expression, as well as its potential role in the immune response. Furthermore, we identify *ACE2, FURIN* and *TMPRSS2* variants and analyze their frequency in different populations (namely, comparing them to the Egyptian population). We also study possible correlations between the population frequencies of the aforementioned variants and indicators of mortality and transmission of SARS-CoV-2 within the same populations.

## 2. Materials and Methods

### 2.1. Analysis of FURIN Gene Expression Patterns in Human Lungs

The LungMAP database (http://www.lungmap.net, accessed on 12 March 2021) currently contains more than 6000 high-resolution lung images, as well as multi-omics data [19]. We used LungMAP to analyze and investigate the gene expression patterns of the FURIN protease in human lung development.

Public RNA-Seq expression data, archived in the NCBI Gene Expression Omnibus (GEO) with accession number (GSE155286) (https://www.ncbi.nlm.nih.gov/geo/query/acc.cgi?acc=GSE155286, accessed on 12 March 2021), were retrieved and analyzed using the DESeq2 R package (1.30.1) [20]. The data were used to analyze the differential expression of *TMPRSS2, ACE2* and *FURIN* in human lungs of SARS-CoV-2-infected human lung-only mice (LoMs) in different time points post-infection—2, 6 and 14 days in comparison to naïve controls [21]. Transcript counts were measured in transcripts-per-million (TPM) and the Z-scores and −log10 values (*p*-values) for each gene in every time point were recorded. Z-scores of Log2 TPMs were visualized in a separate heatmap using the Morpheus tool (https://software.broadinstitute.org/morpheus/, accessed on 12 March 2021).

### 2.2. eQTL Variant Retrieval

To evaluate the relationship between genetic variants and the gene expression profiles of *ACE2, TMPRSS2* and *FURIN*, the expression quantitative trait loci (eQTL) of the three genes were examined using the Genotype–Tissue Expression (GTEx) portal database (http://www.gtexportal.org/home, accessed on 12 March 2021) [22]. TMPRSS2 genetic variants in human lung tissue were obtained from the GTEx portal database. Annotations, such as location or type of variant, were retrieved from the Ensembl Genome Browser (https://www.ensembl.org/index.html, accessed on 12 March 2021) [23].

### 2.3. Retrieval of Population and Clinical Indicator Data

The genetic data and allele frequencies among African, East Asian and European (non-Finnish) populations were extracted from the GnomAD repository, which includes data on a total of 125,748 exomes and 71,702 genomes (https://gnomad.broadinstitute.org/, accessed on 12 March 2021) [24]. Similar Egyptian population data were retrieved from (https://www.egyptian-genome.org/, accessed on 12 March 2021) [25].

Furthermore, in order to study the relationship between eQTL frequencies and indicators of COVID-19 mortality and transmission, indicator data corresponding to case fatality ratio (CFR) percentages, the number of deaths per 100,000 people, and the number of cases per million people were retrieved from (https://coronavirus.jhu.edu/data/mortality, accessed on 12 March 2021) and (https://www.worldometers.info/coronavirus/#countries, accessed on 12 March 2021). Clinical indicators were further studied in relation to eQTL frequencies in ten different populations of available eQTL frequency data in the GnomAD repository, namely, Korea, Japan, Egypt, Finland, Estonia, Bulgaria, Sweden, South America, Africa and Europe. Statistical analysis was conducted using GraphPad Prism V 8.0.2 and allele frequencies for different populations were compared using the Chi-squared test with Yates’s correction. *p*-values are presented as non-corrected values for multiple testing (*p* < 0.05).

### 2.4. Pathway Analysis of ACE2, FURIN and TMPRSS2 Interactome

Pathway Studio Software (version 11.0; Ariadne Genomics/Elsevier Inc., Rockville, MD, USA) was used to deduce relationships among differentially expressed proteomics protein candidates, using the Ariadne ResNet database [26,27]. The significance of the studied proteins was statistically assessed by means of a *t*-test, with *p*-values ≤ 0.05 with a fold change of >1.5 or <0.5. The “Subnetwork Enrichment Analysis” (SNEA) algorithm was selected to extract statistically significant altered biological and functional pathways pertaining to each identified set of protein hits. SNEA utilizes Fisher’s statistical test to determine if there are nonrandom associations between two categorical variables, organized by specific relationships. SNEA starts by creating a central “seed” from all relevant entities in the database, and retrieving associated entities based on their relationship with the seed (that is, binding partners, expression targets, protein modification targets, regulation). The algorithm compares the sub-network distribution to the background distribution using one-sided Mann–Whitney U-test, and calculates a *p*-value indicating the statistical significance of the difference between two distributions. Yellow rectangles, violet rectangles and orange hexagons reflect biological processes, disease processes and functional classes, respectively.

## 3. Results

### 3.1. Gene Expression Analysis in Human Lung Using the LungMAP Database

**Figure 1:**
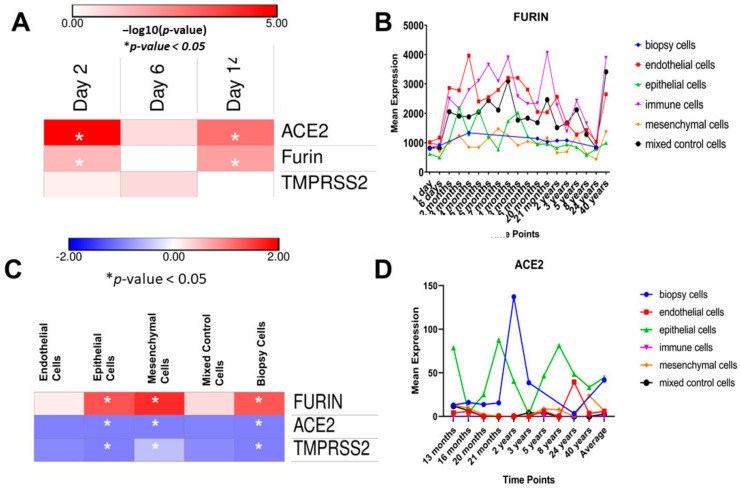
(**A**) −log10 (*p*-value) for the TMPRSS2, ACE2 and FURIN expression levels in the human lungs of naïve human lung-only mice (LoMs) and SARS-CoV-2-infected LoMs, analyzed using expression data retrieved from the NCBI GE× archive (GSE155286). (**B**) RNA-Seq expression level trends of FURIN at different developmental stages in lung cells. (**C**) compares the fold change in the age-dependent expression of FURIN, ACE2 and TMPRSS2 in immune cells versus other cell types, using a two-tailed paired *t*-test. (**D**) RNA-Seq expression level trends of ACE2 at different developmental stages in lung cells.

We analyzed the RNA-Seq differential expression of the (GSE155286) dataset, which compares gene expression in the human lungs of SARS-CoV-2-infected human lung-only mice (LoMs) at different time points post-infection to gene expression naïve controls. We found significant upregulation of ACE2 at day 2 post-infection (log2FC of 2.177817 and a *p*-value of 9.22 × 10^−6^). The differential expression analysis also showed the upregulation of ACE2 TMPRSS2 and FURIN at day 16 post-infection, as shown in the heatmap in Figure 1A. We concluded that the characteristic patterns of expression of *FURIN*, *ACE2* and *TMPRSS2* might provide important insights into the dynamics of SARS-CoV-2 infection.

Furthermore, the analysis of subject genes on the LungMAP website demonstrated that *FURIN* gene expression levels were significantly higher in immune cells than in epithelial, mesenchymal and biopsy cell types (Figure 1B,C). *FURIN* gene expression in immune cells was highest in 21-month-old babies. In epithelial cells, expression was higher in 5-month-old babies and in young children compared to adults. *FURIN* expression was also higher in 40-year-olds than in 24-year-olds and nearly 30 times higher than *ACE2* expression (Figure 1B,D).

### 3.2. Population-Based Frequency Analysis of the Three Studied Genes

**Figure 2:**
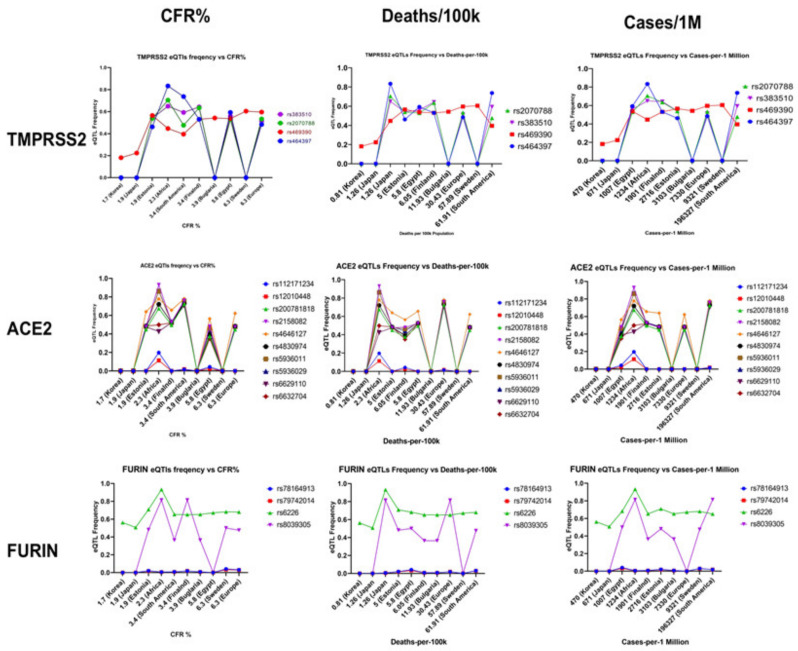
Graphical relationships between frequencies of eQTL variants of TMPRSS2 (first row), ACE2 (second row) and FURIN (third row) in comparison with clinical indicators of mortality (case fatality ratio (CFR) in the first column and deaths per 100,000 people in the second column), as well as cases per million in the third column.

Population allele frequencies for *TMPRSS2, FURIN* and *ACE2* eQTLs in the Egyptian population in statistical comparison with each of the following populations: Africa, East Asia and non-Finnish European are summarized in Table 1. Evaluating the possible patterns of the relationship between eQTL frequencies and indicators of COVID-19 mortality (Figure 2), we found that countries with lower percentages of COVID-19 cases, CFR and deaths, such as Korea and Japan, tend to have lower frequencies of *TMPRSS2, ACE2* and *FURIN* eQTL variants, as shown in the first row of Figure 2. Moreover, eQTL variant rs469390 of TMPRSS2 scored a strong Pearson correlation coefficient of 0.68, with a significant *p*-value of 0.02 at *p* < 0.05, in correlation with the percentages of CFR in the studied populations. Additionally, specific *ACE2* eQTL variants (rs2158082, rs4646127, rs4830974 and rs5936011) (Second row, Figure 2) show overlapping patterns of allele frequencies in the studied populations in relation to mortality ratios and percentages of reported cases. Two eQTL variants of *FURIN* (rs6226 and rs8039305) (Third row, Figure 2) also showed comparable frequency patterns to values of studied indicators, showing relatively higher eQTL frequencies with countries with higher cases and/or mortality ratios, such as Swedish and South American populations. However, significant correlations could not be observed with other variants or in correlation with other indicators studied.

### 3.3. Population-Based Frequencies of FURIN eQTL Variants

To analyze the distribution of *FURIN* eQTLs in lung tissue, we used the GTEx database (https://www.gtexportal.org/home/datasets, accessed on 12 March 2021) (Table 2). Three eQTL variants were found to be associated with *FURIN* expression in lung tissue (rs78164913, rs79742014 and rs8039305). The rs78164913 TT genotype was associated with higher expression compared to the TG genotype, and the CC genotype of rs79742014 was also associated with higher *FURIN* expression in lung tissue compared with the CT genotype. Finally, the the TC genotype of rs8039305 was found to be associated with higher *FURIN* expression in lung tissues, compared with the CC and TT genotypes. The three aforementioned lung eQTL variants were found to be significantly more frequent in the Egyptian population in comparison to the EAS population (*p* < 0.001).

We found the rs6226 GG genotype variant to be significantly less frequent in the Egyptian population compared to the EAS population (*p* = 0.022). We found only one missense FURIN variant covered in the Egyptian-genome study data [28]. rs148110342 was absent in the EAS population and reported with a low AF in the Egyptian population (AF = 0.014). However, the difference in AF in both populations was significant (*p* < 0.001).

### 3.4. Population-Based Frequencies of TMPRSS2 eQTLs in the Lungs

#### 3.4.1. Frequencies of Regulatory Intronic Variants of TMPRSS2

To identify eQTLs associated with *TMPRSS2* expression in the lung tissue, the GTEx portal database was utilized. We identified a total of 203 eQTLs for *TMPRSS2* in all tissues. Among them, 136 variants indicated predominant effects on *TMPRSS2* expression in the lungs (Table S1). Only SNPs associated with clinical significance from the previous literature were considered for further analysis, namely, rs2070788, rs383510 and rs464397. Both rs2070788 GG and GG genotypes of rs383510 variants were found to be relatively more frequent in the Egyptian-genome study data (28) (AF = 0.528 and 0.56, respectively). The rs464397 TT genotype was associated with higher expression of TMPRSS2 in lung tissues and the variant has been found to be more frequent in the Egyptian population (28), with an allele frequency of 0.593, in relation to African and East-Asian populations (*p* = 0.0028 and *p* < 0.0001, respectively).

#### 3.4.2. Frequencies of Common Exonic Variants of TMPRSS2

Our analysis also focused on common exonic variants of *TMPRSS2*; three SNPs showed significantly (*p* < 0.0002) different frequencies when comparing the Egyptian population with the East Asian population (rs2298659, rs17854725 and rs12329760) (*p* = 0.0002, *p* < 0.0001 and *p* < 0.0001 respectively) (Table 1). After investigating their frequencies in the Egyptian-genome study data [28], we found that variant rs28401567 is significantly less frequent in the Egyptian population in comparison to the East-Asian population (*p* ≤ 0.0001).

### 3.5. Population-Based Frequencies of ACE2 Coding, eQTLs and Intronic Variants

We investigated two common *ACE2* exonic variants (rs2285666 and rs35803318). We found that rs2285666 has a relatively lower allele frequency in the Egyptian population in comparison to East-Asians (*p* < 0.0001). Furthermore, we found the variant rs35803318 to be totally absent from the East-Asian population, with a very low allele frequency in the Egyptian population (AF = 0.00935). We investigated the frequency of five common intronic variants (rs2106809, rs4646142, rs714205, rs17264937, and rs5980163) and found that these five variants are relatively less frequent in the Egyptian population than in the East-Asian Population (*p* < 0.0001).

**Figure 3:**
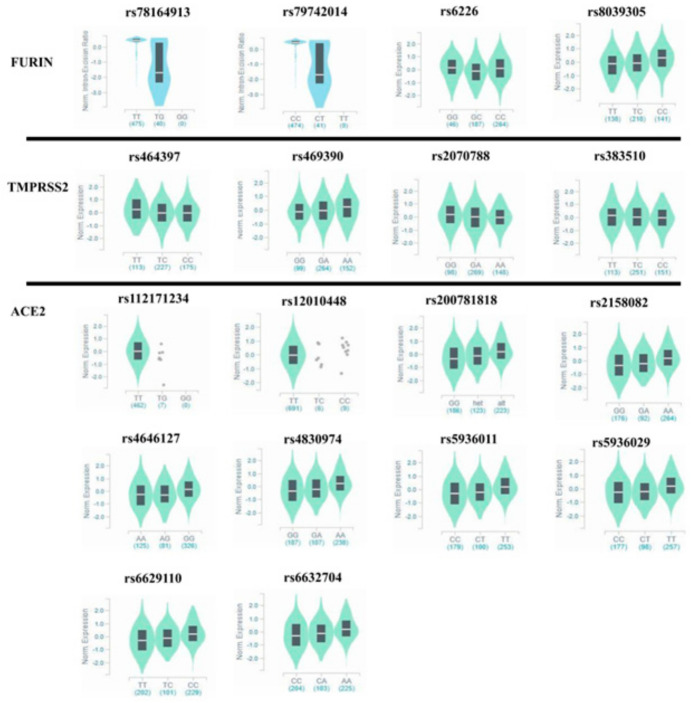
Violin plots representing cis-expression for genotypes of each eQTL SNP for *FURIN*, *TMPRSS2* and *ACE2*.

To analyze the distribution of eQTLs for *ACE2*, we used the Genotype-Tissue Expression (GTEx) database (https://www.gtexportal.org/home/datasets, accessed on 12 March 2021) (Table S3). Fifteen unique eQTL variants (14 SNPs and one INDEL) for ACE2 have been identified, as previously reported by (Cao et al.) [29] with *p*-values ≤  0.05 in 20 tissues from the GTEx database. By investigating the frequency of these eQTL variants in the Egyptian-genome study data, we found that the eQTL variants rs112171234 and rs12010448 showed significantly higher frequency in the Egyptian population compared to the EAS population (*p* < 0.001). However, the following eQTLs (rs200781818, rs2158082, rs4646127, rs4830974, rs5936011, rs5936029, rs6629110 and rs6632704) showed significantly higher frequency in the EAS population compared to other populations, including the Egyptian population (*p* < 0.001) (Table 1, Figure 3).

We also found that eQTL variants rs112171234 and rs12010448 show significantly higher frequency in the Egyptian population compared to the EAS population (*p* < 0.001). However, the following eQTLs (rs200781818, rs2158082, rs4646127, rs4830974, rs5936011, rs5936029, rs6629110 and rs6632704) showed significantly higher frequency in the EAS population compared to other populations [29], including the Egyptian population (*p* < 0.001) (Table 1). We found that *ACE2* variant rs2285666 has a relatively lower allele frequency in the Egyptian population in comparison to East-Asians (*p* < 0.0001). Variant rs35803318 was totally absent for the East-Asian population, it was found to have a very low AF in the Egyptian population as well (AF = 0.00935). We also reported that the five common intronic variants of *ACE2* (rs2106809, rs4646142, rs714205, rs17264937 and rs5980163) have been relatively less frequent in the Egyptian population than the East Asian Population (*p* < 0.0001).

### 3.6. Pathway Analysis and Interaction Network of ACE2, TMPRSS2 and FURIN

**Figure 4:**
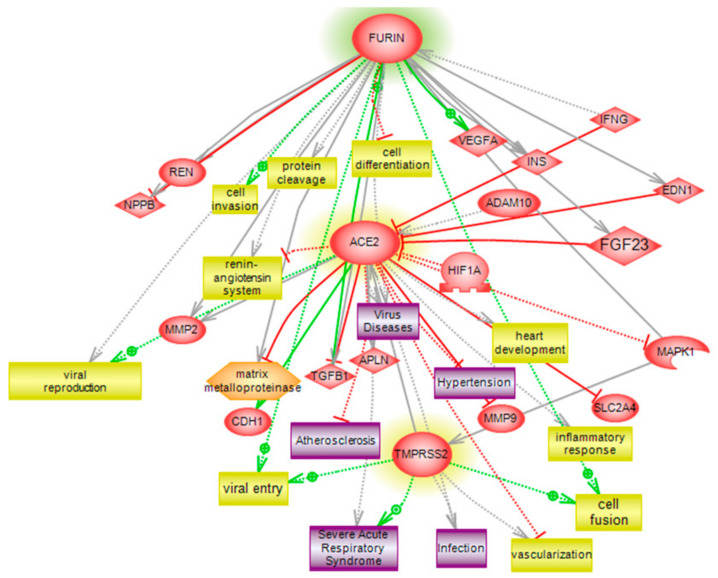
Global Interaction Map and regulatory pathways of the ACE2, FURIN and TMPRSS2 interactome. Identified proteins are highlighted in light pink, showing direct regulatory relationships, including binding, post-translational modifications and transcriptional regulation. Differential pathways were generated using the “direct interaction” algorithm to map interactions and relationships. Based on biological process analysis and molecular functions, these proteins are implicated, including FURIN, in cell differentiation, protein cleavage and cell invasion. Unsupervised pathway assessment showed that TMPRSS2 is implicated in several biological pathways, involving cell fusion, viral entry and vascularization, in addition to being associated with severe acute respiratory syndrome pathogenesis, along with ACE2, which was found to be an upstream regulator for several of the identified interactomes involved in viral reproduction and the inflammatory response to viral infections. In silico validation included the protein entities and biological processes involved, along with the interaction types and directionality, along with the PubMed references utilized to derive these interaction types (Tables S1–S3).

**Figure 5:**
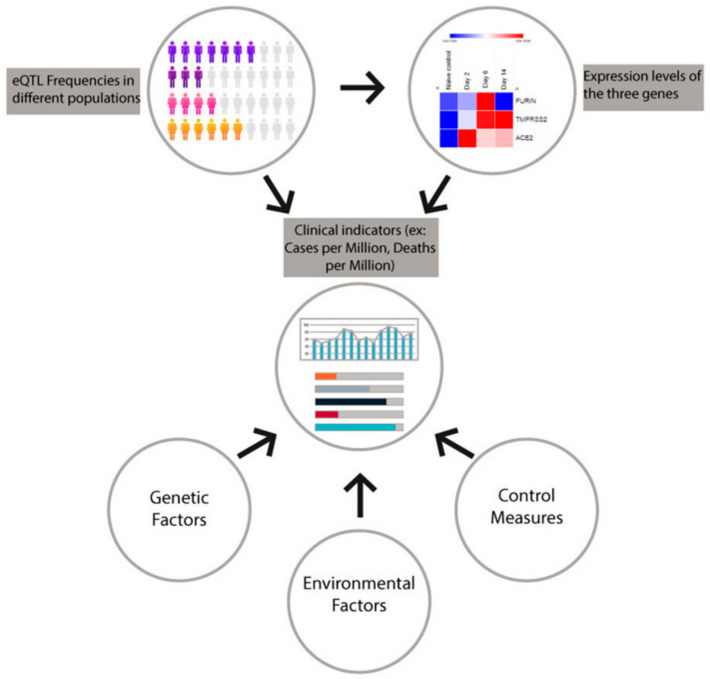
The contribution of eQTL variant frequency, gene expression levels and preventive control measures to clinical indicators of mortality and the transmission of SARS-CoV-2 among different populations.

For the assessment of interactions and pathways, differential pathways were generated using the “direct interaction” algorithm to map the relationships between ACE2, TMPRSS2 and FURIN. We found that FURIN had a direct regulatory function, including in cell differentiation, protein cleavage and cell invasion. Unsupervised pathway assessments showed that TMPRSS2 is implicated in several biological pathways involving cell fusion, viral entry and vascularization, in addition to being associated with severe acute respiratory syndrome pathogenesis, along with ACE2 (Figure 4). Of interest, the protein network showed a centrality relation with ACE2, being an upstream regulator for several of the identified interactomes involving viral reproduction and the inflammatory response to viral infections (Figure 5). The statistical significance of the interaction was determined in silico for the validation process. A detailed depiction of these data is presented in Supplementary File (Tables S1–S3), describing protein entities and biological processes involved, along with the interaction types, directionality and the PubMed reference utilized to derive these interaction types.

## 4. Discussion

### 4.1. Shedding Light on FURIN Expression Patterns and Immune Responses in Relation to SARS-CoV-2 Infection

In this paper, we examined the expression of FURIN as an essential cleavage protease for SARS-CoV-2 cellular entry at the spike glycoprotein S1/S2 cleavage site [30] in light of previous reports of ACE2 and TMPRSS2 expression at different developmental stages of lung tissues [31]. We reported that *FURIN* expression shares similar patterns with *ACE2* and *TMPRSS2* in the epithelial cells of lung tissue. Similarly to ACE2 expression in lung epithelial cells, FURIN recorded the highest expression levels in 5-month-old babies. Additionally, FURIN expression showed a similar pattern of relatively higher expression in the adult alveolar epithelium, compared to that of TMPRSS2, in relation to ACE2 expression [17]. This may provide further evidence that there are significant differences in susceptibility to SARS-CoV-2 among different developmental stages, in addition to the WHO’s general assumption that everyone is deemed susceptible [32], including older age (*p* < 0.0001) [33]. We also found the highest expression levels of FURIN to be in immune cells.

FURIN has been reported to be essential for peripheral immune tolerance [34] and cell-mediated immunity [35]. However, many pathogenic processes could potentially exploit the proprotein convertase FURIN and other convertases [36], for instance, FURIN has been found to be important for the cleavage of various viral glycoproteins, rendering the targeted cells entry-accessible [37,38,39,40,41,42]. Of note, FURIN cleavage of Ebola glycoprotein GP has been suspected to produce a decoy antigen (Ebola GP1), that could structurally induce the apoptosis of uninfected T lymphocytes [43]. As coronaviruses also exploit this cleavage protease [36], SARS-CoV-2 has been reported to depend on FURIN cleavage at S1/S2 as an essential mechanism for S-glycoprotein-mediated cellular fusion and entry [30]. FURIN has also been found to exhibit anti-cancer properties, some of which have been related to adaptive immunity in various cancers [44,45,46]. FURIN activity was found to be vital for cytotoxic T lymphocytes’ expression of immune checkpoints [47,48]. Intriguingly, FURIN inhibition in CD8+ T cells was found to prevent CTL exhaustion and reduce programmed death protein – 1 (PD-1) expression [34]. Additionally, FURIN inactivation has been associated with compromised T regulatory (T reg) functions and increased effector T Cell aggressiveness [49].

Despite exhibiting different roles in different cancer models, FURIN has been suggested as a potential therapeutic target for selective inhibition in different cancers and infectious diseases that exploit the cleavage protease. As SARS-CoV-2 infection has been found to exploit FURIN cleavage, one of the possible implications that should be of interest for further research is the clinical lymphopenia associated with COVID-19 cases [50]. Currently, no evidence of SARS-CoV-2 replication in lymphocytes is available, however, SARS-CoV has been shown to directly infect T lymphocytes, leading to lymphopenia, as well as degeneration of the lymphoreticular system [51]. It is worth investigating whether these clinical manifestations are due to a non-reproductive infection of T lymphocytes, exploiting the proprotein convertase FURIN. Thus, we recommend further research on the association between FURIN cleavage and clinical lymphopenia exhibited by COVID-19 patients.

### 4.2. Implications of Population-Based Variations of Studied Genes’ eQTL Frequencies

#### 4.2.1. FURIN

This study focused on three eQTL variants and found them to be associated with variations in FURIN expression in lung tissues (rs78164913, rs79742014 and rs8039305). Their frequency in the Egyptian population was significantly different to that in the EAS population (*p* < 0.001). One particular eQTL variant, the rs6226 GG genotype, was associated with higher FURIN expression compared to GC and CC genotypes. It is worth mentioning that the former variant has not been found to be associated with hypercholesterolemia in the Kazakh general population [52]. We also investigated a set of previously reported [17] common and rare missense mutations of FURIN, of which only one variant (rs148110342) was absent in the EAS population and showed a low but significantly different AF in the Egyptian population. We consider the differences in AF of both rs8039305 and rs6226 to be intriguing and this may indicate that they might be implicated in the variation of TMPRSS2 expression levels in different populations, warranting further functional investigation.

#### 4.2.2. TMPRSS2

We found the rs2070788 GG genotype to be linked to the higher expression of TMPRSS2 in lung tissues, as well as the GG genotype of rs383510. Both eQTL variants were reported by Cheng at al. to be significantly associated with susceptibility to A(H7N9) and severe A(H1N1)pdm09 influenza in humans [53]. Of note, the haplotype in which rs2070788 and two other SNPs (rs9974589 and rs7364083) are inherited was previously predicted to be associated with higher TMPRSS2 expression [54]. In this study, we linked the rs2070788 GG genotype to higher expression of TMPRSS2 in lung tissues, as well as GG genotype of rs383510, and found both eQTL variants to be relatively more frequent in the Egyptian population, with AF = 0.528 and AF = 0.56, respectively.

Interestingly, rs464397 has been reported to be associated with poor immune responses in patients co-infected with HIV and HCV [55]. We found the rs464397 TT genotype to be associated with higher expression of TMPRSS2 in lung tissues, with a higher frequency in the Egyptian-genome study data [25], with AF = 0.593 in relation to African and East-Asian populations (*p* = 0.0028 and *p* < 0.0001, respectively). Three SNPs showed significantly (*p* < 0.0002) different frequencies when comparing the Egyptian population with the East Asian population (rs2298659, rs17854725 and rs12329760) (*p* = 0.0002, *p* < 0.0001 and *p* < 0.0001 respectively); only rs12329760 was found to be a missense substitution. This variant affects a residue far from the serine protease catalytic triad and was previously found to be significantly associated with TMPRSS2 rearrangements linked to the risk of prostate cancer [56] and a relatively shorter time to diagnosis for high-risk patients [57]. Interestingly, Lopera Maya et al. [58] recently reported TMPRSS2 variants that might be linked to some quantitative phenotypes related to SARS-CoV-2 infection. The strongest associations reported were rs150965978 and rs28401567. The latter is associated with thrombocyte counts that might be linked to clinical thrombocytopenia encountered in COVID-19 patients [59]. We also found that variant rs28401567 is significantly less frequent in the Egyptian population in comparison to the East-Asian population (*p* ≤ 0.0001) (Table 1, Figure 3).

#### 4.2.3. ACE2

Recently, the potential contribution of common exonic variants of *ACE2* to SARS-CoV-2 infection susceptibility at the cellular receptor level has been debated. The variant rs2285666 has been previously tagged as a potential risk factor for coronary artery disease, hypertension and diabetes mellitus [60,61]. Moreover, Wu et al. found a significant association between increases in *ACE2* expression and the AA genotype of the variant [62]. Although the current GTEx dataset does not provide any association between the variant and *ACE2* expression, it is worth investigating its frequency. The variant may be related to *ACE2*- and SARS-CoV-2-associated comorbidities, including hypertension, diabetes and chronic obstructive pulmonary disease [63].

Five common non-coding variants (rs2106809, rs4646142, rs714205, rs17264937 and rs5980163) were previously investigated in SARS-CoV and showed no association with SARS-CoV susceptibility, outcome or prognosis in male patients [64]. Among this set of SNPs, rs2106809 and rs4646142 have been previously reported to be associated with essential hypertension (EH) and carotid arteriosclerosis stenosis (CAS) [65]. Our analyses have shown these variants to be less frequent in the Egyptian population than the EAS population; it may be of interest to explore their potential involvement or lack thereof in SARS-CoV-2 infection and outcomes.

### 4.3. Possible Contribution of eQTL Frequencies to COVID-19 Patterns of Transmission and Mortality; the Bigger Picture

Our analyses of eQTL frequencies showed strong inter-population variations in the frequency of FURIN, TMPRSS2 and ACE2 variants. Although the variants reported both in this study and in other literature might contribute to COVID-19-associated mortality and transmission, it is important to note that such relationships should be studied in the context of other factors, such as control measures implemented, gene expression profiles and population dynamics, as shown in Figure 5. With regards to the molecular landscape of the three studied genes, an abundance of literature is available documenting their interactions with other major cellular pathways, as well as potential therapeutic strategies.

TMPRSS2 might promote viral spread and pathogenesis by facilitating cellular fusion of the SARS-CoV viruses through cathepsin-independent cleavage of the spike glycoprotein. This implication is also supported by the fact that TMPRSS2 has been shown to diminish SARS-CoV recognition by antibodies [66,67]. The ERK/MAPK signaling pathway has been shown to be responsible for optimal induction of TMPRSS2 through both ERK1 and ERK2 [68] and TMPRSS2 expression was sensitive to the loss of expression of ERK2 or ERK1. Thus, the MAPK pathway could also be used for TMPRSS2 targeting [69]. Interestingly, Shirato et al. showed that simultaneous inhibition of TMPRSS2 and cathepsin L has blocked virus entry in-vitro [70]. Thus, the targeting of both cell surface and cathepsin L endosomal entry pathways have been reported as potential treatment strategies for COVID-19 patients [71,72].

Soluble ACE2 was previously shown to exhibit protective effects against severe acute respiratory syndrome as well, probably through catalytically inactive saturation of spike glycoproteins [73,74]. Furthermore, ACE2 overexpression inhibits inflammation, cell growth and VEGFa production in vitro [75]. FURIN expression was also shown to be associated with VEGF-C and TGFß-1 (Figure 4) expression, which creates a feed-forward loop, leading to the enhancement of FURIN expression, which is in turn responsible for cleavage of the aforementioned proteins [76]. FURIN inhibition showed increased secretion of VEGF [77]. In addition, FURIN inhibitors have been shown to block the spike cleavage of SARS-CoV-2, suppressing viral production. Thus, FURIN targeting strategies may be promising interventions for COVID-19 patients [78].

Furthermore, it has been shown that SARS-CoV infection was associated with downregulation of ACE2. Thus, different studies have shown the potential of soluble ACE2 and ACE2 antibodies in the prevention of spike-driven infection [73]. ACE2 deficiency was also associated with the overexpression of inflammatory mediators, including TNF-a, matrix metalloproteinases MMP-9 and interleukin-6 (IL-6). Of note, these mediators could be used as early indicators of COVID-19 infection [79]. Similarly, IL-4 and IFN-gamma have been shown to downregulate the cell surface expression of ACE2 [80]. Furthermore, IL-1beta and TNF alpha have been shown to induce the acute release of ACE2, which supports the protective role of ACE2 against spike-driven infection and ADAM10 regulation of ACE2 cleavage [81]. However, the upregulation of inflammatory mediators, including IFN-γ, has induced the upregulation of FURIN proteins in vitro [82]. Thus, ACE2 could be exploited as well for the pharmacological treatment of COVID-19 [83].

### 4.4. Study Limitations and Future Recommendations

The regulation of *ACE2*, *TMPRSS2* and *FURIN* gene expression is critical for the pathogenesis of SARS-CoV-2 infection. Our study explored the genetic variants responsible for this regulation network. Our study relied mainly on the computational analysis of eQTL frequencies and gene expression levels and was limited by a number of factors. Although the study of said frequencies might provide insight into the trends of transmission and clinical presentation of COVID-19 in different populations, there is a plethora of other factors that should be taken into account to reach plausible conclusions, as explained in Figure 1. Furthermore, we relied on massive genomic data from different general populations, not COVID-19-patient-specific data. Of note, lung-tissue eQTL variants for *ACE2* have not been reported so far in the GTEx browser, which necessitates further investigation into this knowledge gap. In summary, further investigation into the genetic and non-genetic factors affecting COVID-19 infection, transmissibility and outcomes is direly needed both on the virus- and host-level. As more of this information comes to light, it could potentially guide treatment decisions and explain current epidemiological trends.

## 5. Conclusions

We have shed light on FURIN protease expression levels in lung tissues at different developmental stages in light of the expression patterns of the ACE2 receptor and TMPRSS2 protease. We also investigated the frequency of eQTL variants, governing the expression of TMPRSS2, FURIN and ACE2 in the Egyptian population in comparison with other populations. These insights might be helpful in understanding and estimating the genetic factors associated with the transmission of SARS-CoV-2 in different populations and provide insights into the expression patterns of FURIN protease as an essential cleavage protease of SARs-CoV-2.

## Figures and Tables

**Table 1:** Allele frequencies of quantitative trait locus results for TMPRSS2, FURIN and ACE2 in the Egyptian population in statistical comparison with each of the following populations: Africa, East Asia and non-Finnish European.

RSID	Gene	REF	ALT	AF	AC/AN (Egypt)	Africa	East Asia	European	*p*-Value	NES	Tissue
rs78164913	*FURIN*	T	G	0.041	9	50	0	479	1.30 × 10^−31^	−1.6	Lung
					220	8710	1558	15,420			
						<0.0001	<0.0001	0.5416			
rs79742014	*FURIN*	C	T	0.032	7	48	0	445	3.30 × 10^−26^	−1.4	Lung
					220	8712	1560	15,418			
						<0.0001	<0.0001	0.9603			
rs6226	*FURIN*	G	C	0.682	150	23,180	10,584	87,076	0.000008	0.14	Esophagus—Mucosa
					220	24,860	19,918	128,088			
						0.0037	0.022	0.9799			
rs8039305	*FURIN*	T	C	0.5	109	7074	261	7305	1.70 × 10^−17^	0.23	Esophagus—Mucosa
					218	8688	1554	15,370			
						<0.0001	<0.0001	0.7117			
rs464397	*TMPRSS2*	T	C	0.593	128	7250	1554	7458	0.00001	−0.089	Lung
					216	8696	1558	15,392			
						0.0028	<0.0001	0.0826			
rs469390	*TMPRSS2*	G	A	0.537	117	11,136	5216	76,812	0.0000068	0.091	Lung
					218	24,930	19,938	128,862			
						0.1244	<0.0001	0.3904			
rs2070788	*TMPRSS2*	G	A	0.528	114	6125	1034	8207	8.90 × 10^−9^	−0.11	Lung
					216	8694	1556	15,392			
						0.0155	0.0682	0.9765			
rs383510	*TMPRSS2*	T	C	0.56	121	5635	1051	7908	1.20 × 10^−8^	−0.11	Lung
					216	8672	1560	15,350			
						0.2161	0.14	0.5			
rs112171234	*ACE2*	T	G	0.043	9	1159	0	7	0.000041	−0.85	Adipose—Visceral (Omentum)
					210	5882	1003	10,875			
						<0.0001	<0.0001	<0.0001			
rs12010448	*ACE2*	T	C	0.023	4	651	0	2	0.000098	0.43	Muscle—Skeletal
					174	5757	1009	10,497			
						0.0008	<0.0001	<0.0001			
rs200781818	*ACE2*	G	GGGCGCGGTCCTTACGTGT	0.374	74	3582	785	4492	2.00 × 10^−16^	0.27	Nerve—Tibial
					198	5312	836	10,036			
						<0.0001	<0.0001	0.2122			
rs2158082	*ACE2*	G	A	0.481	103	5409	970	5041	1.00 × 10^−16^	0.28	Nerve—Tibial
					214	5816	972	10,610			
						<0.0001	<0.0001	0.9632			
rs4646127	*ACE2*	A	G	0.563	107	3834	858	5756	7.50 × 10^−9^	0.2	Nerve—Tibial
					190	4926	858	9249			
						0.0097	<0.0001	0.4479			
rs4830974	*ACE2*	G	A	0.392	76	4219	952	5140	3.70 × 10^−17^	0.27	Nerve—Tibial
					194	5859	1004	10,670			
						<0.0001	<0.0001	0.1462			
rs5936011	*ACE2*	C	T	0.461	95	4788	936	4951	5.20 × 10^−16^	0.27	Nerve—Tibial
					206	5560	940	10,334			
						<0.0001	<0.0001	0.8084			
rs5936029	*ACE2*	C	T	0.441	90	4761	927	4921	1.40 × 10^−14^	0.26	Nerve—Tibial
					204	5504.00	931.00	10,198.00			
						<0.0001	<0.0001	0.52			
rs6629110	*ACE2*	T	C	0.39	79.00	2459	952.00	4996.00	3.40 × 10^−15^	0.25	Nerve—Tibial
					202.00	5772.00	989.00	10,571.00			
						0.57	<0.0001	0.18			
rs6632704	*ACE2*	C	A	0.35	74.00	2868	944.00	5088.00	4.60 × 10^−16^	0.26	Nerve—Tibial
					210.00	5757.00	992.00	10,604.00			
						0.01	<0.0001	0.03			

**Table 2:** Allele frequencies of common exonic and intronic regulatory variants of TMPRSS2, FURIN and ACE2 in the Egyptian population in statistical comparison with each of the following populations: Africa, East Asia and non-Finnish European.

RSID	Gene	REF	ALT	AF	AC/AN (Egypt)	Africa	East Asia	European	Amino Acids	SIFT	POLYPHEN
rs148110342	*FURIN*	C	T	0.014	3	4	0	206	p.Arg81Cys	0.04	0.59
					216	24,902	19,940	128,808			
						<0.0001	<0.0001	0.0003			
rs150965978	*TMPRSS2*	C	A	0.004545	1	83	0	765	Intron Variant		
					220	8706	1560	15,408			
						0.6913	0.2541	0.0046	Intron Variant		
rs28401567	*TMPRSS2*	C	T	0.241	53	2885	1147	3161	-		
					220	8696	1552	15,404			
						0.0446	<0.0001	0.3371			
rs2298659	*TMPRSS2*	G	A	0.125	27	4592	5179	28,744	p.Gly259Gly	synonymous variant	synonymous variant
					216	23,850	19,478	122,880			
						0.0415	0.0002	0.0024			
rs17854725	*TMPRSS2*	A	G	0.427	93	9066	2544	67,712	p.Ile256Ile	synonymous variant	synonymous variant
					218	23,918	19,604	122,814			
						0.3754	<0.0001	0.0438			
rs12329760	*TMPRSS2*	C	T	0.188	41	7265	7651	29,831	p.Val160Met	0	0.938
					218	24,896	19,934	128,604			
						0.0118	<0.0001	0.2485			
rs3787950	*TMPRSS2*	T	C	0.185	40	5000	2905	9864	p.Thr75Thr	synonymous variant	synonymous variant
					216	24,832	19,600	127,666			
						0.6889	0.2324	<0.0001			
rs2285666	*ACE2*	C	T	0.15600	34.00000	4057.00000	7336.00000	17,240.00000	c.439+4G>A	intron variant	splice region variant
					218.00000	18,323.00000	13,387.00000	86,164.00000			
						0.06890	<0.0001	0.20460			
rs35803318	*ACE2*	C	T	0.00935	2.00000	133.00000	0	3935.00000	p.Val749Val	non-coding transcript exon variant	non-coding transcript exon variant
					214.00000	18,549.00000	13,918.00000	88,946.00			
						0.97210	<0.0001	0.02470			
rs2106809	*ACE2*	A	G	0.15000	32.00000	707.00000	533.00000	1938.00000	intron variant		
					214.00000	5771.00000	954.00000	10,584.00000			
						0.35390	<0.0001	0.33090			
rs4646142	*ACE2*	G	C	0.15200	32.00000	1372.00000	557.00000	2162.00000	intron variant		
					210.00000	5792.00000	979.00000	10,704.00000			
						0.0257	<0.0001	0.16410			
rs714205	*ACE2*	C	G	0.13200	28.00000	707	544.00000	1927.00000	intron variant		
					212.00000	5865	969.00000	10,714.00000			
						0.73400	<0.0001	0.15010			
rs17264937	*ACE2*	T	C	0.16000	32.00000	289.00000	382.00000	3041.00000	intron variant		
					200.00000	5698.00000	946.00000	10,493.00000			
						<0.0001	<0.0001	0.00220			
rs5980163	*ACE2*	C	G	0.02400	5.00000	13.00000	0.00000	128.00000	intron variant		
					212.00000	5887.00000	997.00000	10,850.00000			
						<0.0001	<0.0001	0.22400			

## Data Availability

Not Applicable.

## Supplementary Materials

The following are available online at https://www.mdpi.com/article/10.3390/genes12071041/s1, Table S1: Entity Table; Table S2: Interaction Table and References; Table S3: Interactions and COVID-19 pathways.

## Abbreviations

**Term**	**Definition**
SARS-CoV-2	severe acute respiratory syndrome coronavirus-2
MERS	Middle East respiratory syndrome
ACE2	angiotensin-converting enzyme 2
TMPRSS2	transmembrane protease, serine 2
eQTL	expression quantitative trait loci are genomic loci that explain variations in the expression levels of mRNA transcripts.
GEO	Gene Expression Omnibus
LoM	lung-only mice
GTEx	Genotype-Tissue Expression portal database
“GC”, “CC” Genotypes of rs-id of a variant	the nucleotides represent the genotype of a specific eQTL variant, labeled with its unique rs-id.
CFR	case fatality ratio
TPM	transcripts-per-million
