# Supplementary material for: Bioinformatics Analysis of Allele Frequencies and Expression Patterns of ACE2, TMPRSS2 and FURIN in Different Populations and Susceptibility to SARS-CoV-2
Source: Genes (Basel). 2021 Jul 5;12(7):1041. doi: 10.3390/genes12071041 (PMC8303858; doi:10.3390/genes12071041)
Supplement: Supplementary file 1 [file genes-12-01041-s001.zip › genes-1215509-supplementary.pdf]

**Supplementary File-1: Gene Interaction Analysis**
**Table-1: Entity Table**

| Name | Type | Description | Connectivity | Local Connectivity | Indegree |
|---|---|---|---|---|---|
| CDH1 | Protein | cadherin 1, type 1, E-cadherin (epithelial) | 2700 | 1 | 1 |
| SLC2A4 | Protein | solute carrier family 2 (facilitated glucose transporter), member 4 | 1361 | 1 | 1 |
| HIF1A | Protein | hypoxia inducible factor 1, alpha subunit (basic helix-loop-helix transcription factor) | 3259 | 1 | 0 |
| FURIN | Protein | furin (paired basic amino acid cleaving enzyme) | 731 | 22 | 3 |
| FGF23 | Protein | fibroblast growth factor 23 | 439 | 2 | 1 |
| VEGFA | Protein | vascular endothelial growth factor A | 6072 | 1 | 1 |
| IFNG | Protein | interferon, gamma | 6096 | 2 | 0 |
| INS | Protein | insulin | 8218 | 2 | 2 |
| NPPB | Protein | natriuretic peptide B | 1250 | 2 | 2 |
| APLN | Protein | apelin | 764 | 1 | 1 |
| EDN1 | Protein | endothelin 1 | 3337 | 2 | 1 |

| | | | | | |
|---|---|---|---|---|---|
| TGFB1 | Protein | transforming growth factor, beta 1 | 7286 | 3 | 2 |
| MAPK1 | Protein | mitogen-activated protein kinase 1 | 7201 | 3 | 1 |
| MMP2 | Protein | matrix metallopeptidase 2 (gelatinase A, 72kDa gelatinase, 72kDa type IV collagenase) | 2728 | 2 | 2 |
| MMP9 | Protein | matrix metallopeptidase 9 (gelatinase B, 92kDa gelatinase, 92kDa type IV collagenase) | 3554 | 1 | 1 |
| REN | Protein | renin | 1369 | 1 | 1 |
| ADAM10 | Protein | ADAM metallopeptidase domain 10 | 624 | 1 | 0 |
| ACE2 | Protein | angiotensin I converting enzyme 2 | 567 | 24 | 6 |
| TMPRSS 2 | Protein | transmembrane protease, serine 2 | 100 | 7 | 1 |
| Hyperte nsion | Disease | | 1029 | 1 | 1 |
| cell different iation | Cell Process | | 7022 | 1 | 1 |
| inflamm atory response | Cell Process | | 2351 | 1 | 1 |
| vasculari zation | Cell Process | | 3045 | 2 | 2 |
| Atherosc lerosis | Disease | | 1508 | 1 | 1 |
| renin- angioten | Cell Process | | 258 | 2 | 2 |

| | | | | | |
|---|---|---|---|---|---|
| sin system | | | | | |
| Infection | Disease | | 2524 | 2 | 2 |
| matrix metalloproteinase | Functional Class | | 3516 | 2 | 2 |
| viral reproduction | Cell Process | | 1449 | 2 | 2 |
| cell invasion | Cell Process | | 1987 | 1 | 1 |
| heart development | Cell Process | | 1029 | 1 | 1 |
| viral entry | Cell Process | | 458 | 2 | 2 |
| Virus Diseases | Disease | | 956 | 2 | 2 |
| cell fusion | Cell Process | | 407 | 2 | 2 |
| Severe Acute Respiratory Syndrome | Disease | | 79 | 2 | 2 |
| protein cleavage | Cell Process | | 57 | 1 | 1 |

## Table-2: Interaction Table and References

| Relation | Organ | Type | Sentence | TextRef | Connectivity | # of References | Owner | Found In Pathways |
|---|---|---|---|---|---|---|---|---|
| TMPRSS2 ---> vascularization | | Regulation | TMPRSS2 may play a role in angiogenesis and tubulogenesis in microvesicular endothelial cells, potentially modulating several aspects of prostate tumor biology. | info:pmid/20382709#body:6 | 2 | 1 | | covid |
| FURIN ---> viral reproduction | Liver {Organ urn:agi-ncimorgan:CL384198} | Regulation | Our results show that furin, thrombospondin-1, and TGF-ß1 positively regulate Hepatitis C virus replication., Our results showed poor hepatitis B e antigen secretion after furin inhibition, which may lead to the mis-incorporation of hepatitis B e antigen P22 proprotein into the hepatitis B virus nucleocapsid polymer, alter the structure of the hepatitis B virus nucleocapsid, and decrease the efficacy of hepatitis B virus replication and HBsAg biosynthesis. | info:pmid/21296375#body:192, info:pmid/22634051#body:87 | 2 | 2 | | Neighbors of viral reproduction |

| | | | | | | | | |
|---|---|---|---|---|---|---|---|---|
| FURIN ---> protein cleavage | | Regulation | Since activation of PAR1 quickly initiates the internalization and degradation of the receptor [78] we hypothesize that the presence of unactivated PAR1 at the cell surface inhibits furin-mediated F protein cleavage., Taken together, these results suggest that the correlation between the cellular furin abundance and the productive infectious bronchitis virus infection in different cells may be due to a differential efficiency of S protein cleavage mediated by furin. | info:pmid/24015257#cont:221, info:pmid/22995191#body:97 | 2 | 2 | | |
| FURIN --+> viral entry | | Regulation | Detailed time course experiments showed that a peptide furin inhibitor, decanoyl-Arg-Val-Lys-Arg-chloromethylketone, blocked both viral entry and syncytium formation., As furin-mediated cleavage of S protein at the second furin site was shown to play an important role in viral infectivity by promoting virus-cell and cell-cell fusion ( ), it would be reasonable to speculate that furin may facilitate viral entry and spread. | info:pmid/19553314#abs:3, info:pmid/22995191#body:135 | 2 | 2 | | |
| MAPK1 ---> TMPRSS2 | | Expression | Interestingly, we found that TMPRSS2 expression was sensitive to loss of either ERK2 or ERK1; this androgen-regulated promoter is linked to portions of the ..., | info:pmid/18787043#body:253, info:pmid/19463689#body:95 | 2 | 2 | | covid |

| | | | | | | | | |
|---|---|---|---|---|---|---|---|---|
| | | | Moreover, only ERK2 is required for optimal androgen dependent induction of PSA, but optimal induction of TMPRSS2 requires both ERK1 and ERK2. | | | | | |
| TMPRSS2 ---> ACE2 | | ProtModification | Shulla and colleagues demonstrated that TMPRSS2, HAT and TMPRSS 11a cleave coexpressed ACE2 and cleavage was associated with increased infectivity ( )., Upon contact of ACE2 with S protein, ACE2 is also cleaved by TMPRSS2 [ ]. | info:pmid/24121034#body:158, info:doi/10.1016/j.coviro.2011.05.014#body:14 | 2 | 2 | | |
| IFNG ---> FURIN | | Regulation | In HepG2.2.15 cells, interferon- > further suppressed furin and hepatitis B e antigen expression., We confirmed by immunoblot that PMA, LPS, and IFN-? induce the upregulation of furin protein in THP-1 cells ( B). | info:pmid/22634051#abs:8, info:pmid/24138882#body:63 | 2 | 2 | | |
| FURIN --+> VEGFA | | MolTransport | Treatment with a furin inhibitor reduced the secretion efficiency of the VEGF, indicating that furin digestion increases the secretion of VEGF., The relative VEGF secretion was ~600% in the absence of the furin inhibitor and decreased to ~400% in the presence of 10μmol of the furin inhibitor. | info:pmid/22450332#abs:9, info:doi/10.1016/j.jconrel.2012.03.010#body:112 | 2 | 2 | | |
| IFNG ---\| ACE2 | | Expression | Consistent with these observations, IL-4 and IFN-gamma downregulated cell surface expression of angiotensin-converting enzyme 2 (ACE2), the severe | info:pmid/16860835#abs:5, info:pmid/20599443#body:82 | 2 | 2 | | |

| | | | | | | | | |
|---|---|---|---|---|---|---|---|---|
| | | | acute respiratory syndrome coronavirus receptor., Consistently, Interferon-? and interleukin-4 downregulate expression of ACE2 mRNA levels in epithelial cells ( ). | | | | | |
| FURIN ---> renin-angiotensin system | | Regulation | Previous research indicates that the FURIN gene may play a pivotal role in the renin-angiotensin system and maintaining the sodium-electrolyte balance., FURIN can directly regulate the renin-angiotensin system and factors that maintain the sodium-electrolyte balance [113]. | info:pmid/20707915#abs:3, info:pmid/24396277#cont:203 | 2 | 2 | | |
| ADAM10 ---> ACE2 | airway {Organ urn:agi-ncimorgan:C0458827} | Regulation | Phorbol ester, ionomycin, endotoxin, and IL-1beta and TNFalpha acutely induced ACE2 release, further supporting that ADAM17 and ADAM10 regulate ACE2 cleavage., Immunoblotting revealed that the mixed ADAM10/ADAM17 inhibitor (GW280264X) effectively blocked stimulated ACE2 shedding at low concentrations, whereas the ADAM10-selective inhibitor (GI254023X) had a much lower potency (Fig. 4). | info:pmid/19411314#abs:6, info:pmid/15983030#body:167 | 2 | 2 | | |

| | | | | | | | | |
|---|---|---|---|---|---|---|---|---|
| MAPK1 ---> FURIN | | Expression | However, in the presence of T3, furin activation is modulated by MEK/ERK., ... transforming growth factor-ß1 and is regulated by it.4 Even though proprotein convertase5 and PC7 have several structural, biochemical, and cell biological similarities to furin, transforming growth factor-ß1 did not increase the levels of proprotein convertase5 or PC7 mRNAs.4 In vascular endothelial cells, furin increases with increased fluid shear stress, whereas proprotein convertase5 remains unaffected.25 Furthermore, studies in other cell lines report ERK-mitogen-activated protein kinase ... | info:pmid/18467449#body:414, info:pmid/11882580#body:163 | 2 | 2 | | |
| FURIN ---> REN | | ProtModification | In contrast, the nanobodies could inhibit only the furin-mediated processing of renin and not that of other PCs., As shown in Fig. 1, renin cleaved from the prorenin-furin precursor was predominant in the 2-h labeling supernatants, suggesting that prosegment cleavage occurred in the constitutive secretory pathway. | info:pmid/22920187#cont:188, info:pmid/8702811#body:129 | 2 | 2 | | |
| ACE2 ---> inflammatory response | | Regulation | We hypothesized that ACE2 overexpression may inhibit inflammation response in atherosclerotic plaque by degrading Ang II into Ang-(1-7)., In atherosclerosis-prone apolipoprotein E knockout | info:pmid/19961735#abs:2, info:pmid/21099686#abs:4 | 2 | 2 | | |

| | | | | | | | | | |
|---|---|---|---|---|---|---|---|---|---|
| | | | mice, ACE2 deficiency results in augmented vascular inflammation and an inflammatory response that contributes to increased atherosclerotic plaque formation. | | | | | | |
| ACE2 ---\| SLC2A4 | | Expression | The expression of GLUT4 and MEF2A was increased by angiotensin 1-7 in ACE2 knockout mice and decreased by A779 in wild-type mice., (1) demonstrated that regulation of myocyte enhancer factor 2A and GLUT4 expression by the ACE type 2/angiotensin 1–7/Mas receptor axis contribute to the improvement of insulin sensitivity. | info:pmid/22933108#abs:7, info:pmid/23801722#cont:13 | 2 | 2 | | | |
| HIF1A ---\| ACE2 | Pulmonary artery {Organ urn:agi-ncimorgan:C0034052}, Brain {Organ urn:agi-ncimorgan:C1269537} | Regulation | Thus HIF-1alpha inhibited ACE2 expression, and the accumulated ANG II catalyzed by ACE is a key mediator in the downregulation of ACE2 by HIF-1alpha., One potential mechanism may involve transcriptional repression of ACE2 by hypoxia-inducible factor 1a or activation by hepatocyte nuclear factor 1ß (29,30). | info:pmid/19592460#abs:9, info:pmid/22289845#cont:395 | 2 | 2 | | | |
| ACE2 --+> CDH1 | Kidney {Organ urn:agi-ncimorgan:C1278978} | Expression | This is the first study to elucidate the mechanism through which the overexpression of ACE2 in the A549 lung cancer cell line decreases metastasis formation in vivo and upregulates the expression of E-cadherin both in vitro and | info:pmid/23545945#abs:6, info:pmid/21189404#cont:447 | 2 | 2 | | | |

| | | | | | | | | |
|---|---|---|---|---|---|---|---|---|
| | | | in vivo., angiotensin II–mediated renal fibrosis was also associated with decreased membrane-fractionated E-cadherin protein levels consistent with epithelial-to-mesenchymal transition, which was prevented by recombinant human ACE2 (Supplemental Figure SIV, available online at http://hyper. ahajournals.org). | | | | | |
| ACE2 --+> viral reproduction | | Regulation | Together, these observations suggest that the affinity of S protein for ACE2 is an important determinant in the overall rate of viral replication and in the severity of disease., Replication of the virus in cells and the formation of syncitia can be blocked by antibodies against ACE2, which indicates the importance of ACE2 in the replication of Severe Acute Respiratory Syndrome-CoV ( )., Importantly, syncytia formation/membrane fusion and viral replication can be specifically inhibited by an anti-angiotensin-converting enzyme-2 antibody ( ) or a fragment containing the receptor binding domain ( ) or antibodies recognizing the receptor binding domain ( ). | info:pmid/15791205#body:246, info:pmid/15464852#body:99, info:pmid/15708633#body:39 | 2 | 3 | | |

| | | | | | | | | | |
|---|---|---|---|---|---|---|---|---|---|
| FURIN ---> MMP2 | | ProtModification | Furin-cleaved MMP-2 does not possess proteolytic activity as examined in a cell-free assay., Furin can activate matrix metalloproteinases and cleave pro-MMP-2 or -9 to MMP-2 or -9 (37, 38)., of MMP-2 was used as a positive control for furin activity, as MMP-2 is activated by furin target MMP-14, and is cleaved by furin as well.27 | info:pmid/15637056#abs:5, info:pmid/18467449#body:298, info:pmid/22330140#cont:266 | 2 | 3 | | | |
| FURIN ---\| NPPB | | Expression | If furin converts ?BNP to BNP-45, a higher level of furin expression may result in the increased production of BNP-45., Furin and corin seem to be involved in the degradation of proBNP into BNP 1-32 and NT-proBNP 1-76 within or during secretion from the cardiomyocyte., Prohormone convertases such as furin and corin seem to be involved in the degradation of pro-B-type natriuretic peptide into BNP 1-32 and NT-pro-B-type natriuretic peptide 1-76 within or during secretion from the cardiomyocyte. | info:pmid/9001393#body:123, info:pmid/23470072#cont:64, info:pmid/19147726#body:81 | 2 | 3 | | | |

| | | | | | | | | |
|---|---|---|---|---|---|---|---|---|
| FURIN ---> INS | | Expression | Having determined that the cell lines produced insulin in response to glucose, we asked if production of mature insulin in the engineered cells was uniquely dependent on furin activity, which is produced in glucose-regulated manner and ultimately is responsible for production of mature insulin., The furin sites allow a ubiquitous endopeptidase to process proinsulin made in the non-ß-cells, facilitating the secretion of mature insulin, the biologically active form of the peptide, to facilitate the production and secretion of mature insulin in human non-ß-cells. <more data available...> | info:pmid/17920636#body:135, info:pmid/16403445#body:27, info:pmid/20719072#cont:203 | 2 | 3 | | |
| ACE2 ---\| matrix metalloproteinase | Heart {Organ urn:agi-ncimorgan:C1281570}, Aorta {Organ urn:agi-ncimorgan:C1278934} | Expression | More intriguingly, recombinant human ACE2 treatment significantly abolished AngII-mediated increases in MMP2, MMP9 and MT1-matrix metalloproteinase in cardiac fibroblasts., Overexpression of ACE2 inhibits invasion and Matrix metalloproteinase production in vitro., Along with this concept, Thomas et al12 now show that ACE2 knockout mice have increased aortic expression of matrix metalloproteinases-2 and matrix metalloproteinases-9, as well as increased matrix | info:pmid/24161906#abs:8, info:pmid/21769437#cont:160, info:pmid/20884883#cont:47 | 2 | 3 | | |

| | | | | | | | | |
|---|---|---|---|---|---|---|---|---|
| | | | metalloproteinases-9 production in macrophages. | | | | | |
| FGF23 ---\| ACE2 | Kidney {Organ urn:agi-ncimorgan:C1278978} | Expression | Finally, we found that FGF23 suppresses angiotensin-converting enzyme 2 (ACE2) expression in the kidney, thereby providing a pathway for FGF23 regulation of the renin-angiotensin system., First, there is evidence that FGF23 may regulate the renin angiotensin system via suppression of ACE2 expression in the kidney., In this regard, FGF23 appears to be a potent inhibitor of ACE2 expression in the kidney (11), which prevents degradation of Ang I and Ang II. | info:pmid/22970174#abs:7, info:pmid/23465500#body:162, info:pmid/23298840#cont:30 | 2 | 3 | | |
| FURIN ---> Virus Diseases | | Regulation | Novel furin inhibitors or modified forms of D6R may promote the reduction of immune tolerance and the elimination of infected hepatocytes in patients with chronic hepatitis B virus infection., Various studies have confirmed that furin plays a crucial role in many bacterial and viral diseases, tumorigenesis, neurodegenerative disorders and diabetes., Therefore, furin inhibitors hold great | info:pmid/23617302#abs:10, info:pmid/21168329#body:3, info:pmid/17179036#body:53, info:pmid/12832286#body:85 | 2 | 4 | | Neighbors of Virus Diseases |

| | | | | | | | | |
|---|---|---|---|---|---|---|---|---|
| | | | promise as potential therapeutic agents for treating furin-mediated diseases and viral and bacterial infections, particularly for short-term therapy. <more data available...> | | | | | |
| TMPRSS2 --+> cell fusion | | Regulation | Directed protease expression and inhibition analyses revealed that TMPRSS2 and endosomal cathepsins activate EMC-S for virus-cell fusion and constitute potential targets for antiviral intervention., Indeed, engineered expression of TMPRSS2 and HAT rendered 229E S-protein-driven virus-cell fusion insensitive to an inhibitor of cathepsin L, a protease previously shown to facilitate human coronavirus 229E infection., However, TMPRSS2 but not human airway trypsin-like protease expression rendered SARS-S-driven virus-cell fusion independent of cathepsin activity, indicating that human airway trypsin-like protease and TMPRSS2 activate SARS-S differentially. <more data available...> | info:pmid/23468491#abs:6, info:pmid/23536651#abs:5, info:pmid/21994442#abs:7, info:pmid/24121034#body:113 | 2 | 4 | | covid |

| | | | | | | | | | |
|---|---|---|---|---|---|---|---|---|---|
| TMPRSS2 --+> viral entry | | Regulation | Thus, TMPRSS2 affects the entry of virus but not other phases of virus replication., Simultaneous treatment with inhibitors of cathepsin L and TMPRSS2 completely blocked virus entry into Vero-TMPRSS2 cells, indicating that Middle East respiratory syndrome coronavirus employs both the cell surface and the endosomal pathway to infect Vero-TMPRSS2 cells., Gierer et al. used VSV-luciferase pseudotyped with the Middle East respiratory syndrome coronavirus S to determine that Middle East respiratory syndrome coronavirus does not utilize any other coronaviral receptors, that TMPRSS2 and endosomal cathepsins facilitate viral entry, and that the S protein can be neutralized with Middle East respiratory syndrome coronavirus infected patient serum ( ). <more data available...> | info:pmid/20926566#abs:6, info:pmid/24027332#abs:6, info:pmid/24269477#body:157, info:pmid/24473083#cont:461 | 2 | 4 | | | |

| | | | | | | | | |
|---|---|---|---|---|---|---|---|---|
| TMPRSS2 ---> Infection | Lung {Organ urn:agi-ncimorgan:C1278908} | Regulation | These observations suggest camostat as a candidate antiviral drug to prevent or depress TMPRSS2-dependent infection by severe acute respiratory syndrome coronavirus., Accordingly, coexpression of the 1918 hemagglutinin with TMPRSS4 or the previously identified hemagglutinin-processing protease TMPRSS2 allowed trypsin-independent infection by pseudotypes bearing the 1918 hemagglutinin, indicating that these proteases might support 1918 influenza virus spread in the lung., In a recent study, it was described that the inhibition of TMPRSS2 by the non-specific serine protease inhibitor camostat caused a 10-fold reduction in infection of Calu-3 cells by SARS-CoV [19]. <more data available...> | info:pmid/22496216#abs:6, info:pmid/19158246#abs:8, info:pmid/23527573#cont:51, info:pmid/24121034#body:167 | 2 | 4 | | covid |
| FURIN --+> cell fusion | | Regulation | Furin activity is required for BeWo cell fusion in vitro., A) Inhibition of syncytin 1- and syncytin 2-mediated cell fusion by furin inhibitor I., Finally, we showed that CREB mediated furin activation was critical during trophoblast cell fusion process. | info:pmid/23598405#cont:43, info:pmid/18650494#body:196, info:embase/2013417921#cont:43, info:doi/10.1016/j.placenta.2012.06.016#body:3663 | 2 | 4 | | |

| | | | | | | | | |
|---|---|---|---|---|---|---|---|---|
| ACE2 ---> MMP2 | Heart {Organ urn:agi-ncimorgan:C1281570}, Aorta {Organ urn:agi-ncimorgan:C1278934} | Expression | The specific mechanisms by which ACE2 regulates MMP-2, however, remain unclear., More intriguingly, recombinant human ACE2 treatment significantly abolished AngII-mediated increases in MMP2, MMP9 and MT1-matrix metalloproteinase in cardiac fibroblasts., MMP-2 activity ( B1 and B2) and MMP2 protein expression level ( C1 and C2) were significantly increased in the Ad-ACE2 group in comparison with the Ad-EGFP and mock groups., Deficiency in ACE2 increases the aortic expression of pro-inflammatory mediators including TNF-a, interleukin-6 (IL-6), MCP-1, vascular cell adhesion molecule 1 (VCAM-1), MMP-2 and MMP-9 , which is accompanied by an increase in adhesion of leukocytes to ECs in vitro and to blood vessel ex vivo . | info:pmid/24219285#abs:3, info:pmid/24161906#abs:8, info:pmid/22340266#body:51, info:pmid/22947420#body:82 | 2 | 4 | | |
| ACE2 ---> Virus Diseases | | Regulation | Peptides representing various regions of ACE2 critical for virus infection were chemically synthesized and evaluated for antiviral activity., Although the idea is less clear and somewhat controversial, Severe acute respiratory syndrome-coronavirus is thought to use C-type lectins DC-SIGN and/or L-SIGN (collectively referred to as DC/L-SIGN) as | info:pmid/16510163#abs:5, info:pmid/17715238#abs:3, info:pmid/18490652#body:47, info:pmid/16033974#body:153, info:pmid/18 | 2 | 5 | | |

| | | | | | | | | |
|---|---|---|---|---|---|---|---|---|
| | | | alternative receptors or as enhancer factors that facilitate ACE2-mediated virus infection., In addition, siRNAs of TACE and ACE2 blocked viral infection., We then investigated whether a known ACE2-specific peptide inhibitor competed against ACE2-mediated pseudotype virus infection. <more data available...> | 801550#body:72 | | | | |
| TMPRSS2 --+> Severe Acute Respiratory Syndrome | | Regulation | In summary, we show that TMPRSS2 might promote viral spread and pathogenesis by diminishing viral recognition by neutralizing antibodies and by activating SARS S for cell-cell and virus-cell fusion., The type II transmembrane protease TMPRSS2 activates the spike (S) protein of severe acute respiratory syndrome coronavirus on the cell surface following receptor binding during viral entry into cells., However, TMPRSS2 but not human airway trypsin-like protease expression rendered SARS-S-driven virus-cell fusion independent of cathepsin activity, indicating that human airway trypsin-like protease and TMPRSS2 activate SARS-S differentially. <more data available...> | info:pmid/21325420#abs:7, info:pmid/22496216#abs:1, info:pmid/21994442#abs:7, info:pmid/22222211#body:81, info:pmid/23527573#cont:49 | 2 | 5 | | covid |

| | | | | | | | | |
|---|---|---|---|---|---|---|---|---|
| ACE2 ---\| vascularization | Aorta {Organ urn:agi-ncimorgan:C1278934} | Regulation | These results suggest that the overexpression of ACE2 may potentially suppress the invasion and angiogenesis of non-small cell lung cancer., Here, we show, both in vitro and in vivo, that ACE2 inhibited the development of early atherosclerotic lesions by suppressing the growth of vascular smooth muscle cells and improving endothelial function., A recent study showed that ACE2 could inhibit the angiogenesis of non-small cell lung cancer by decreasing VEGF ., Similarly, recent evidence suggests that the overexpression of ACE2 may influence angiogenesis by inhibiting inflammation, cell growth, and VEGFa production in vitro . <more data available...> | info:pmid/21769437#abs:8, info:pmid/20798044#abs:3, info:pmid/21481527#body:125, info:pmid/22749485#body:78, info:pmid/22947420#body:68 | 2 | 5 | | |
| FURIN ---> FGF23 | Kidney {Organ urn:agi-ncimorgan:C1278978} | ProtModification | By this model, furin-mediated FGF23 processing would be increased in the iron-deficient state to guard against hypophosphatemia., Furin, a subtilisin-like proprotein convertase enzyme, cleaves intact FGF-23 and generates smaller N-terminal (18 kDa) and C-terminal (12 kDa) fragments (27)., The site of incorporation was determined by MALDI-TOF analysis of the furin-cleaved product of the FGF23b GalNAc glycopeptide, where the N-terminal peptide | info:pmid/22921867#body:122, info:pmid/22573526#cont:245, info:pmid/16638743#body:161, info:pmid/12506157#body:219, info:pmid/20837471#cont:238 | 2 | 5 | | |

| | | | | | | | | |
|---|---|---|---|---|---|---|---|---|
| | | | fragment was found as the expected mass (calculated mass 1032.2) plus the saccharide component (calculated mass 1235) (Fig. 3). <more data available...> | | | | | |
| ACE2 ---\| MMP9 | Heart {Organ urn:agi-ncimorgan:C1281570}, Aorta {Organ urn:agi-ncimorgan:C1278934} | Expression | More intriguingly, recombinant human ACE2 treatment significantly abolished AngII-mediated increases in MMP2, MMP9 and MT1-matrix metalloproteinase in cardiac fibroblasts., We also found that the overexpression of ACE2 suppressed A549 cell invasion and MMP-2 and MMP-9 activity in vitro and in vivo., In our study, genetic Ace2 deficiency was associated with increased aortic expression of gelatinases, matrix metalloproteinase-2 (gelatinase-A) and MMP-9 (gelatinase-B), and augmented production of MMP-9 in macrophages. <more data available...> | info:pmid/24161906#abs:8, info:pmid/21769437#cont:217, info:pmid/20671240#cont:234, info:pmid/20798044#cont:215, info:pmid/22947420#body:82, info:pmid/18403726#body:195 | 2 | 6 | | |

| | | | | | | | | |
|---|---|---|---|---|---|---|---|---|
| EDN1 ---\| ACE2 | Bronchi {Organ urn:agi-ncimorgan:C0006255}, Heart {Organ urn:agi-ncimorgan:C1281570} | Expression | Endothelin-1 (ET-1) also significantly reduced myocyte ACE2 mRNA., ET-1 downregulates ACE2 expression and activity at the transcription level in human bronchial epithelial cells via the endothelin A receptor by a p38 mitogen-activated protein kinase-dependent mechanism., This finding supports results of a previous study whereby ET-1 treatment at 10 nM for 12 h reduced ACE2 mRNA by ~60% in cardiomyocytes (21)., In cardiac myocytes and fibroblasts, ACE-2 mRNA and protein are downregulated by ANGII or endothelin -1 and upregulated by the angiotensin receptor AT1 blocker losartan [26]. <more data available...> | info:pmid/18849338#abs:6, info:pmid/23751363#abs:6, info:pmid/23836146#cont:190, info:pmid/23100504#cont:306, info:pmid/21045683#cont:64, info:pmid/21881224#cont:228 | 2 | 6 | | |
| ACE2 ---> heart development | Heart {Organ urn:agi-ncimorgan:C1281570}, Epicardium {Organ urn:agi-ncimorgan:C0225968}, Blood Vessels {Organ urn:agi-ncimorga | Regulation | Perspectives The present study demonstrated that ACE2 might suppress the development of cardiac hypertrophy and congestive heart failure induced by pressure overload., In support of this possibility is evidence that deletion of the ACE2 gene leads to the development of heart failure and that this effect can be inhibited by further deletion of the ACE gene ( )., A previous study suggested that ACE2 is involved in cardiac development, whereby the | info:pmid/16505206#body:233, info:pmid/18718424#body:25, info:pmid/24177423#cont:154, info:pmid/22523556#cont:186, info:pmid/23249272#cont:183, info:pmid/23608725#body | 2 | 7 | | |

| | | | | | | | | |
|---|---|---|---|---|---|---|---|---|
| | n:C0005847}, Coronary artery {Organ urn:agi-ncimorgan:C1269008} | | ACE2 promoter is activated in epicardial cells in Xenopus embryos via a GATA-binding site (39). <more data available...> | :94, info:doi/10.1016/j.cardfail.2007.06.303#body:1 | | | | |
| FURIN ---> INS | Liver {Organ urn:agi-ncimorgan:CL384198}, Pancreas {Organ urn:agi-ncimorgan:C1278931} | ProtModification | Furin protease cleaved insulin peptides in vitro., Since the biochemical characteristics of the insulin proreceptor processing endopeptidase activity mostly resembled those of furin activity, it is likely that insulin proreceptor proteolytic maturation can be catalysed by furin in the liver., In these experiments a tetracycline-inducible promoter was inserted above the furin-modified insulin coding sequence., Proinsulin cleaved by furin is processed to chromatographically mature insulin by carboxypeptidases in nonneuroendocrine cells. <more data available...> | info:pmid/21143365#abs:10, info:pmid/8037679#abs:11, info:pmid/11593361#body:184, info:pmid/8844768#title:1, info:pmid/12085245#body:59, info:pmid/17920636#body:104, info:pmid/8995442#body:48 | 2 | 7 | | |
| FURIN ---> NPPB | Kidney {Organ urn:agi-ncimorgan:C1278978}, Heart {Organ urn:agi-ncimorgan:C12815 | ProtModification | Previous studies showed that both furin and corin cleaved pro-BNP., There is evidence to suggest that amphibian BNP precursors are probably cleaved by the endoprotease furin, which recognises a specific amino acid sequence Arg-X-X-Arg ( )., In this pathway, the BNP precursor is thought to be cleaved by | info:pmid/21763278#body:64, info:pmid/16343494#body:25, info:pmid/9252368#body:51, info:pmid/9252369#body: | 2 | 8 | | |

| | | | | | | | | |
|---|---|---|---|---|---|---|---|---|
| | 70}, Heart Atrium {Organ urn:agi-ncimorgan:C0018792} | | furin, because the BNP precursor possesses a furin-cleavable RXXR sequence at its processing site (19)., Pro-BNP is cleaved by corin or furin, mainly in the cytoplasm of cardiac myocytes, to yield to N-terminal (NT-proBNP) and C-terminal (BNP) portions of proBNP. <more data available...> | 51, info:pmid/23684562#body:7, info:pmid/15265821#body:146, info:pmid/19147726#body:54, info:doi/10.1016/j.cardfail.2011.06.076#body:1 | | | | |
| ACE2 ---\| MAPK1 | umbilical artery {Organ urn:agi-ncimorgan:C0041632}, Left ventricular {Organ urn:agi-ncimorgan:C0225897}, Aorta {Organ urn:agi-ncimorgan:C1278934}, Heart {Organ urn:agi-ncimorgan:C1281570}, Kidney {Organ | Regulation | More importantly, treatment with human recombinant ACE2 (1mg/ml) dramatically prevented Angiotensin II-mediated SOCS3 expression and the JAK2-STAT3 and ERK1/2 signaling, and resulted in attenuation of superoxide production and cell proliferation in Human umbilical artery smooth muscle cells., Importantly, treatment with telmisartan (1 or 10 µM) or recombinant human ACE2 (2mg/ml) largely ameliorated angiotensin II-induced profilin-1 expression and extracellular-signal regulated kinase 1/2 and JNK phosphorylation and augmented PPAR? ?expression in the cultured human umbilical artery smooth muscle cells. <more data available...> | info:pmid/23816468#abs:7, info:pmid/20854846#abs:7, info:pmid/22595130#body:108, info:pmid/22693641#cont:139, info:pmid/20679547#body:150, info:pmid/24161906#cont:195, info:pmid/21189404#cont:437, info:doi/10.1016/j.peptides.2012.01.020#body:16 | 2 | 8 | | |

| | | | | | | | | |
|---|---|---|---|---|---|---|---|---|
| | urn:agi-ncimorgan:C1278978} | | | | | | | |
| ACE2 ---\| TGFB1 | Kidney {Organ urn:agi-ncimorgan:C1278978}, Cardiovascular system {Organ urn:agi-ncimorgan:C1269562}, Islets of Langerhans {Organ urn:agi-ncimorgan:C0022131} | Expression | After 8weeks of treatment, compared with Goldblatt group, felodipine+puerarin reduced SBP, DBP and HR (p<0.01 or p<0.05), ameliorated renal interstitial fibrosis, decreased the level of Ang II and increased that of Ang (1-7), upregulated mRNA expression of ACE2 and Mas, decreased that of ACE and AT1, and downregulated protein expression of TGF-ß1 in kidneys (p<0.01)., Ang II–mediated myocardial fibrosis and expression of procollagen type Ia1, procollagen type IIIa1, transforming growth factor-ß1, and fibronectin were also suppressed by recombinant human ACE2. <more data available...> | info:pmid/23523569#abs:5, info:pmid/20679547#cont:19, info:pmid/23174757#body:18, info:pmid/22340267#body:29, info:pmid/21189404#cont:635, info:pmid/22340266#body:66, info:pmid/18948167#body:114, info:doi/10.1016/j.cardfail.2010.06.054#body:5 | 2 | 8 | | |

| | | | | | | | | |
|---|---|---|---|---|---|---|---|---|
| FURIN --+> cell invasion | Veins {Organ urn:agi-ncimorgan:C0042449}, Liver {Organ urn:agi-ncimorgan:CL384198}, Endometrium {Organ urn:agi-ncimorgan:C1550633} | Regulation | Furin complementation resulted in an increased cell invasiveness that correlated with their capacity to produce MMP-2., Our results demonstrated that the furin inhibitor decreased pro-MT1-MMP processing as well as pro-MMP-2 activation and cell invasiveness., In contrast, over-expression of furin markedly increased cell invasion and migration (P<0.01), accompanied by significant increase of MMP-9 activities., Furin over-expression could occur in liver cancer and a previous study showed that over-expression of furin promoted HepG2 cell invasion in tail vein xenograft models. <more data available...> | info:pmid/14644155#abs:6, info:pmid/9539163#abs:6, info:pmid/19853298#abs:7, info:pmid/22808247#abs:3, info:pmid/23835774#cont:206, info:pmid/18467449#body:349, info:pmid/16194539#body:124, info:pmid/23598405#cont:27, info:doi/10.1016/S0014-4827(03)00407-5#body:169, info:embase/2013417921#cont:27 | 2 | 10 | | Neighbors of cell invasion |

| | | | | | | | | |
|---|---|---|---|---|---|---|---|---|
| FURIN ---> EDN1 | Lower jaw region {Organ urn:agi-ncimorgan:C0460026}, Bone and Bones {Organ urn:agi-ncimorgan:C0262950}, female reproductive system {Organ urn:agi-ncimorgan:C0700038}, Heart {Organ urn:agi-ncimorgan:C1281570} | ProtModification | Analysis of the processing of precursors of endothelin-1 (ET-1), adrenomedullin , transforming growth factor ß1 (TGF-ß1), and bone morphogenetic protein 4 (BMP4) confirmed that ET-1, adrenomedullin, and TGF-ß1 are in vivo substrates of endothelial furin., These studies suggest that Edn1 is cleaved twice, first by Furin and second by ECE1 and that these cleavages are required for Edn1 bioactivity., Bone morphogenic protein 7 and endothelin-1 were both glycosylated and cleaved by furin in vitro but glycosylation did not yield protection against processing. <more data available...> | info:pmid/22733989#abs:9, info:pmid/17574232#body:17, info:pmid/21937429#cont:293, info:pmid/22553222#cont:82, info:pmid/22406696#body:17, info:pmid/16678149#body:18, info:pmid/23470073#cont:21, info:pmid/11577023#body:72, info:pmid/11067800#body:69, info:doi/10.1016/j.drudis.2012.02.017#body:17 | 2 | 10 | | |

| | | | | | | | | |
|---|---|---|---|---|---|---|---|---|
| ACE2 ---> APLN | Cardiovascular system {Organ urn:agi-ncimorgan:C1269562}, hypothalamus {Organ urn:agi-ncimorgan:C0020663}, Heart {Organ urn:agi-ncimorgan:C1281570} | ProtModification | Apelin is a second catalytic substrate for ACE2 and functions as an inotropic and cardioprotective peptide., Apelin is also a catalytic substrate for angiotensin-converting enzyme 2, the key severe acute respiratory syndrome receptor., ACE2 also cleaves some peptides, such as dynorphin, apelin and bradykinin., ACE2 most efficiently cleaves apelin-13, dynorphin A (1-13), and des-Arg9 bradykinin (4, 5)., Specific cleaving of apelin peptides by ACE2, the first human homologue of angiotensin-converting enzyme (ACE), has been observed ( ). <more data available...> | info:pmid/24177423#abs:2, info:pmid/17673668#abs:2, info:pmid/20700837#cont:78, info:pmid/16166094#body:70, info:pmid/15907343#body:185, info:pmid/11815627#body:220, info:pmid/20605969#cont:73, info:pmid/23962453#body:18, info:pmid/16298469#body:8, info:pmid/21099686#cont:56 <more data available...> | 2 | 12 | | |

| | | | | | | | | |
|---|---|---|---|---|---|---|---|---|
| FURIN ---\| cell differentiation | Liver {Organ urn:agi-ncimorgan:CL384198}, Ectoderm {Organ urn:agi-ncimorgan:C0013574}, Stomach {Organ urn:agi-ncimorgan:C1278920}, Cochlea {Organ urn:agi-ncimorgan:C0009195}, Epidermis {Organ urn:agi-ncimorgan:C0014520} | Regulation | Thus, furin appears to control the proliferation as well as differentiation of islet cells., Thus, we conclude that Interleukin 12 induction of furin might represent a new aspect of IFN-gamma regulation and control of T helper 1 differentiation., The liver produces a number of proproteins having a furin-cleavable site; thus, furin may be involved in growth and differentiation both in the partially hepatectomized liver and in primary cultured hepatocytes., These results indicate that Sty1/Spc1 controls the cellular differentiation process by controlling Atf1. <more data available...> | info:pmid/8895387#abs:12, info:pmid/16627761#abs:6, info:pmid/9426209#abs:2, info:pmid/9585499#body:131, info:pmid/21896659#cont:125, info:pmid/23545100#body:110, info:pmid/9109428#title:1, info:pmid/18713856#body:237, info:pmid/23277081#cont:88, info:pmid/15505202#body:137 <more data available...> | 2 | 13 | | Neighbors of cell differentiation |
| FURIN ---> matrix metalloproteinase | Kidney Glomerulus {Organ urn:agi-ncimorgan:C0022663} | ProtModification | These data indicate that furin can directly cleave Matrix metalloproteinases containing an RXXR motif., Furthermore, the matrix metalloprotease MMP-14, which is PMA-inducible (reviewed in Ref. 34), is cleaved by furin., Indeed, furin was demonstrated to cleave this sequence in | info:pmid/15637056#body:244, info:pmid/19047044#body:307, info:pmid/9037199#body:5, info:pmid/10471791#body | 2 | 13 | | |

| | | | | | | | | |
|---|---|---|---|---|---|---|---|---|
| | | | prostromelysin-3 and proMT1-Matrix metalloproteinase resulting in enzyme activation ., The presence of an RXK/RR motif suggests that the prodomain of CA-matrix metalloproteinase will be cleaved off by furin or related enzymes in the trans-Golgi network. <more data available...> | :150, info:pmid/19166965#body:55, info:pmid/15560752#body:243, info:pmid/17332756#body:52, info:pmid/12665557#body:299, info:pmid/20686912#cont:132, info:pmid/20605060#body:122 <more data available...> | | | | |
| ACE2 ---> Infection | Heart {Organ urn:agi-ncimorgan:C1281570}, Lung {Organ urn:agi-ncimorgan:C1278908} | Regulation | The soluble ectodomain of ACE2 specifically abrogated S-mediated infection and might therefore be exploited for the generation of inhibitors., Finally, we show that a soluble and catalytically inactive form of ACE2 potently blocked infection by S-protein-pseudotyped retrovirus and by severe acute respiratory syndrome-CoV., Pulmonary infection with the human severe acute respiratory syndrome-CoV in mice led to an ACE2-dependent myocardial infection with a marked decrease in ACE2 expression confirming a critical role of | info:pmid/15194496#abs:3, info:pmid/15367630#abs:7, info:pmid/19453650#abs:5, info:pmid/16730806#body:9, info:pmid/16337697#body:13, info:pmid/16510163#body:57, info:pmid/15897467#body | 2 | 16 | | |

| | | | | | | | | |
|---|---|---|---|---|---|---|---|---|
| | | | ACE2 in mediating severe acute respiratory syndrome-CoV infection in the heart. <more data available...> | :115, info:pmid/23962453#body:64, info:pmid/16033974#body:129, info:pmid/15381196#body:65 <more data available...> | | | | |
| FURIN ---> TGFB1 | Salivary Glands {Organ urn:agi-ncimorgan:C0036098}, Bone and Bones {Organ urn:agi-ncimorgan:C0262950}, Respiratory System {Organ urn:agi-ncimorgan:C1269561}, Prostate {Organ urn:agi-ncimorgan:C1278980}, Brain {Organ urn:agi- | ProtModification | Analysis of the processing of precursors of endothelin-1 (ET-1), adrenomedullin , transforming growth factor ß1 (TGF-ß1), and bone morphogenetic protein 4 (BMP4) confirmed that ET-1, adrenomedullin, and TGF-ß1 are in vivo substrates of endothelial furin., TGF-ß positively regulates expression of furin and is a furin substrate (64) ., As with TGFß ligands, the inhibin precursor molecules are apparent substrates for furin cleavage., Furin has traditionally been suggested to cleave pro-TGF-ß intracellularly during processing in the golgi apparatus. <more data available...> | info:pmid/22733989#abs:9, info:pmid/12832286#body:150, info:pmid/18826948#body:54, info:pmid/18826955#body:54, info:pmid/18243766#body:41, info:pmid/15975431#body:224, info:pmid/24098777#cont:72, info:pmid/17516499#title:1, info:pmid/22808247#cont:282, info:pmid/20870411#body | 2 | 20 | | |

| | | | | | | | | |
|---|---|---|---|---|---|---|---|---|
| | ncimorgan:C1269537} | | | :14 <more data available...> | | | | |
| ACE2 ---\| Atherosclerosis | Blood Vessels {Organ urn:agi-ncimorgan:C0005847}, Kidney {Organ urn:agi-ncimorgan:C1278978}, Arteries {Organ urn:agi-ncimorgan:C0003842}, Cerebrum {Organ urn:agi-ncimorgan:C1280654}, Carotid Arteries {Organ urn:agi-ncimorga | Regulation | ACE2 deficiency increased atherosclerotic area (Ace2(+/y), 17 ± 1; Ace2(-/y), 23 ± 2 mm(2), P < 0.002)., However, the underlying mechanisms by which ACE2 effectively suppresses early atherosclerotic lesions remain poorly understood., Angiotensin converting enzyme-2 confers endothelial protection and attenuates atherosclerosis., ACE2 degrades pro-atherosclerotic Ang II and increases anti-atherosclerotic angiotensin 1-7., (2008) Angiotensin converting enzyme-2 confers endothelial protection and attenuates atherosclerosis. <more data available...> | info:pmid/21252069#abs:5, info:pmid/20798044#abs:2, info:pmid/18660448#title:1, info:pmid/22947420#body:141, info:pmid/23249272#cont:351, info:pmid/24147777#cont:265, info:pmid/22659526#cont:350, info:pmid/20599443#body:60, info:pmid/23266545#cont:6, info:pmid/24193738#title: | 2 | 23 | | |

| | | | | | | | | | |
|---|---|---|---|---|---|---|---|---|---|
| | n:C0007272}, Aorta {Organ urn:agi-ncimorgan:C1278934}, Heart {Organ urn:agi-ncimorgan:C1281570} | | | 1 <more data available...> | | | | | |
| ACE2 ---> Severe Acute Respiratory Syndrome | Lung {Organ urn:agi-ncimorgan:C1278908} | Regulation | Importantly, ACE2 has been identified as a key SARS-coronavirus receptor and plays a protective role in SARS pathogenesis., Finally, we show that a soluble and catalytically inactive form of ACE2 potently blocked infection by S-protein-pseudotyped retrovirus and by severe acute respiratory syndrome-CoV., Infection by severe acute respiratory syndrome coronavirus is initiated by specific interactions between the severe acute respiratory syndrome coronavirus spike (S) protein and its receptor ACE2., Monkey and human kidney cells (LLC-MK2, Vero, and 769-P) and swine kidney cells were permissive for both viruses, but only severe acute respiratory syndrome-CoV infection could be blocked by anti-human ACE2 antibody | info:pmid/20134095#abs:4, info:pmid/15367630#abs:7, info:pmid/19853613#abs:1, info:pmid/23232719#abs:8, info:pmid/18554741#body:172, info:pmid/23962453#body:64, info:pmid/15980414#body:65, info:pmid/18801550#body:119, info:pmid/21533129#cont:135, | 2 | 24 | | |

| | | | | | | | | | |
|---|---|---|---|---|---|---|---|---|---|
| | | | and could be neutralized by preincubation of virus with soluble ACE2. <more data available...> | info:pmid/24219285#cont:280 <more data available...> | | | | | |
| TGFB1 --+> FURIN | Aorta {Organ urn:agi-ncimorgan:C1278934}, Synovial Membrane {Organ urn:agi-ncimorgan:C0039099}, Lung {Organ urn:agi-ncimorgan:C1278908}, Heart {Organ urn:agi-ncimorgan:C1281570}, Arteries | Expression | Furthermore, TGF-beta itself increased the furin mRNA levels., Although the furin mRNA level was increased by TGF-beta1 in Dami cells, it was not affected by PDGF-BB., Stimulation with TGFbeta1 results in a significant increase in furin mRNA levels, starting at 3 h with the peak effect observed at 12 h (2.5-fold increase +/- 0.4)., In primary culture of rat hepatocytes, furin expression increases gradually with time, and its expression is greatly enhanced by transforming growth factor beta1, whose processing from the precursor requires cleavage by furin. <more data available...> | info:pmid/11348875#abs:6, info:pmid/12097169#abs:9, info:pmid/9109442#abs:4, info:pmid/9426209#abs:4, info:pmid/21112328#abs:10, info:pmid/18467449#body:423, info:pmid/22808247#cont:286, info:pmid/12832286#body:150, info:pmid/12177061#body:415, info:pmid/17 | 2 | 28 | | |

| | | | | | | | | | |
|---|---|---|---|---|---|---|---|---|---|
| | {Organ urn:agi-ncimorgan:C0003842}, Pulmonary artery {Organ urn:agi-ncimorgan:C0034052}, Cornea {Organ urn:agi-ncimorgan:C1550625}, artery wall {Organ urn:agi-organ:artery%20wall}, Bone and Bones {Organ urn:agi-ncimorgan:C0262950}, Pituitary Gland {Organ urn:agi-ncimorgan:C1278880} <more data | | | 948127#body:106 <more data available...> | | | | | |

| | | | | | | | | |
|---|---|---|---|---|---|---|---|---|
| | available...> | | | | | | | |
| ACE2 ---\| Hypertension | Kidney {Organ urn:agi-ncimorgan:C1278978}, Brain {Organ urn:agi-ncimorgan:C1269537}, Kidney Glomerulus {Organ urn:agi-ncimorgan:C0022663}, hypothalamic paraventricular nucleus {Organ urn:agi-organ:hypothalamic%20paraventricular%20nu | Regulation | ACE2 amplification may have a potential therapeutic role for kidney disease and hypertension., This prevention of hypertension by ACE2 overexpression was reversed by blockade of the Ang-(1-7) receptor (d-Ala7-Ang-[1-7]; 600 ng/kg per minute)., Amplifying angiotensin-converting enzyme 2 activity may have a potential therapeutic role for kidney disease and hypertension., In addition, overexpression of ACE2 in the brain reduces hypertension by improving arterial baroreflex and autonomic function., We hypothesized that ADAM17-mediated ACE2 shedding results in decreased membrane-bound ACE2 in the brain, thus promoting the development of neurogenic hypertension. <more data available...> | info:pmid/18775121#abs:4, info:pmid/19926873#abs:6, info:pmid/18408475#abs:8, info:pmid/19124678#abs:10, info:pmid/24014829#abs:3, info:pmid/21952934#abs:4, info:pmid/17325232#abs:11, info:pmid/16878172#abs:9, info:pmid/23968354#abs:9, info:pmid/18022600#abs:1 | 2 | 62 | | |

| | | | | | | | | |
|---|---|---|---|---|---|---|---|---|
| | cleus}, Medulla Oblongata {Organ urn:agi-ncimorgan:C1269575}, Blood Vessels {Organ urn:agi-ncimorgan:C0005847}, Cardiovascular system {Organ urn:agi-ncimorgan:C1269562}, Heart Ventricle {Organ urn:agi-ncimorgan:C0018827}, Heart {Organ urn:agi-ncimorgan:C1281570}, Coronary artery {Organ urn:agi-ncimorga | | | <more data available...> | | | | |

| | | | | | | | | |
|---|---|---|---|---|---|---|---|---|
| | n:C1269008} <more data available...> | | | | | | | |
| ACE2 ---\| renin-angiotensin system | Kidney {Organ urn:agi-ncimorgan:C1278978}, Heart {Organ urn:agi-ncimorgan:C1281570}, Cardiovascular system {Organ urn:agi-ncimorgan:C1269562}, Brain {Organ urn:agi-ncimorgan:C1269537}, Pancreas {Organ urn:agi- | Regulation | It is a consequence of this action that ACE2 participates in the renin-angiotensin system., Angiotensin-converting enzyme 2 (ACE2) is a new component of the renin-angiotensin system ., Angiotensin-converting enzyme-2 (ACE2) is a negative regulator of the renin-angiotensin system., Angiotensin-converting enzyme 2 (ACE2) is a newly identified component of renin-angiotensin system., Angiotensin-converting enzyme 2 (ACE2) is a newly identified regulator of the renin-angiotensin system., Angiotensin-converting enzyme 2 (ACE2) is an important negative regulator of the renin-angiotensin system. <more data available...> | info:pmid/15549171#abs:4, info:pmid/20424953#abs:1, info:pmid/22474255#abs:2, info:pmid/21769437#abs:2, info:pmid/19759332#abs:1, info:pmid/21285291#abs:1, info:pmid/18307733#abs:2, info:pmid/17703127#abs:1, info:pmid/23706365#abs:1, info:pmid/22 | 2 | 100 | | |

| | | | | | | | |
|---|---|---|---|---|---|---|---|
| | ncimorgan:C1278931}, Lung {Organ urn:agi-ncimorgan:C1278908}, Intestines {Organ urn:agi-ncimorgan:C0021853}, Alveolus {Organ urn:agi-ncimorgan:C1515933}, Vascular system {Organ urn:agi-ncimorgan:C0489903}, Aorta {Organ urn:agi-ncimorgan:C1278934} <more data available...> | | | 800722#abs:4 <more data available...> | | | |

**Table-3: Interactions and COVID-19 pathways**

| Relation | Organ | Type | Sentence | TextRef | Connectivity | # of References | Owner | Found In Pathways |
|---|---|---|---|---|---|---|---|---|
| TMPRSS2 ---> vascularization | | Regulation | TMPRSS2 may play a role in angiogenesis and tubulogenesis in microvesicular endothelial cells, potentially modulating several aspects of prostate tumor biology. | info:pmid/20382709#body:6 | 2 | 1 | | covid |
| FURIN ---> viral reproduction | Liver {Organ urn:agi-ncimorgan:CL384198} | Regulation | Our results show that furin, thrombospondin-1, and TGF-ß1 positively regulate Hepatitis C virus replication., Our results showed poor hepatitis B e antigen secretion after furin inhibition, which may lead to the mis-incorporation of hepatitis B e antigen P22 proprotein into the heptatitis B virus nucleocapsid polymer, alter the structure of the heptatitis B virus nucleocapsid, and decrease the efficacy of heptatitis B virus replication and HBsAg biosynthesis. | info:pmid/21296375#body:192, info:pmid/22634051#body:87 | 2 | 2 | | Neighbors of viral reproduction |

| | | | | | | | | |
|---|---|---|---|---|---|---|---|---|
| FURIN ---> protein cleavage | | Regulation | Since activation of PAR1 quickly initiates the internalization and degradation of the receptor [78] we hypothesize that the presence of unactivated PAR1 at the cell surface inhibits furin-mediated F protein cleavage., Taken together, these results suggest that the correlation between the cellular furin abundance and the productive infectious bronchitis virus infection in different cells may be due to a differential efficiency of S protein cleavage mediated by furin. | info:pmid/24015257#cont:221, info:pmid/22995191#body:97 | 2 | 2 | | |
| FURIN --+> viral entry | | Regulation | Detailed time course experiments showed that a peptide furin inhibitor, decanoyl-Arg-Val-Lys-Arg-chloromethylketone, blocked both viral entry and syncytium formation., As furin-mediated cleavage of S protein at the second furin site was shown to play an important role in viral infectivity by promoting virus-cell and cell-cell fusion ( ), it would be reasonable to speculate that furin may | info:pmid/19553314#abs:3, info:pmid/22995191#body:135 | 2 | 2 | | |

| | | | | | | | | |
|---|---|---|---|---|---|---|---|---|
| | | | facilitate viral entry and spread. | | | | | |
| MAPK1 ---> TMPRSS2 | | Expression | Interestingly, we found that TMPRSS2 expression was sensitive to loss of either ERK2 or ERK1; this androgen-regulated promoter is linked to portions of the ..., Moreover, only ERK2 is required for optimal androgen dependent induction of PSA, but optimal induction of TMPRSS2 requires both ERK1 and ERK2 . | info:pmid/18787043#body:253, info:pmid/19463689#body:95 | 2 | 2 | | covid |
| TMPRSS2 ---> ACE2 | | Prot Modification | Shulla and colleagues demonstrated that TMPRSS2, HAT and TMPRSS11a cleave coexpressed ACE2 and cleavage was associated with increased infectivity ( )., Upon contact of ACE2 with S protein, ACE2 is also cleaved by TMPRSS2 [ ]. | info:pmid/24121034#body:158, info:doi/10. | 2 | 2 | | |

| | | | | | | | | |
|---|---|---|---|---|---|---|---|---|
| | | | | 1016/j.coviro.2011.05.014#body:14 | | | | |
| IFNG ---> FURIN | | Regulation | In HepG2.2.15 cells, interferon-? further suppressed furin and hepatitis B e antigen expression., We confirmed by immunoblot that PMA, LPS, and IFN-? induce the upregulation of furin protein in THP-1 cells ( B). | info:pmid/22634051#abs:8, info:pmid/24138882#body:63 | 2 | 2 | | |
| FURIN --+> VEGFA | | MolTransport | Treatment with a furin inhibitor reduced the secretion efficiency of the VEGF, indicating that furin digestion increases the secretion of VEGF., The relative VEGF secretion was ~600% in the absence of the furin inhibitor and decreased to ~400% in the presence of 10μmol of the furin inhibitor. | info:pmid/22450332#abs:9, info:doi/10.101 | 2 | 2 | | |

| | | | | | | | | |
|---|---|---|---|---|---|---|---|---|
| | | | | 6/j.jconrel.2012.03.010#body:112 | | | | |
| IFNG ---\| ACE2 | | Expression | Consistent with these observations, IL-4 and IFN-gamma downregulated cell surface expression of angiotensin-converting enzyme 2 (ACE2), the severe acute respiratory syndrome coronavirus receptor., Consistently, Interferon-? and interleukin-4 downregulate expression of ACE2 mRNA levels in epithelial cells ( ). | info:pmid/16860835#abs:5, info:pmid/20599443#body:82 | 2 | 2 | | |
| FURIN ---> renin-angiotensin system | | Regulation | Previous research indicates that the FURIN gene may play a pivotal role in the renin-angiotensin system and maintaining the sodium-electrolyte balance., FURIN can directly regulate the renin-angiotensin system and factors that maintain the sodium-electrolyte balance [113]. | info:pmid/20707915#abs:3, info:pmid/24396277 | 2 | 2 | | |

| | | | | | | | | |
|---|---|---|---|---|---|---|---|---|
| | | | | #cont:203 | | | | |
| ADAM10 ---> ACE2 | airway {Organ urn:agi-ncimorgan:C0458827} | Regulation | Phorbol ester, ionomycin, endotoxin, and IL-1beta and TNFalpha acutely induced ACE2 release, further supporting that ADAM17 and ADAM10 regulate ACE2 cleavage., Immunoblotting revealed that the mixed ADAM10/ADAM17 inhibitor (GW280264X) effectively blocked stimulated ACE2 shedding at low concentrations, whereas the ADAM10-selective inhibitor (GI254023X) had a much lower potency (Fig. 4). | info:pmid/19411314#abs:6, info:pmid/15983030#body:167 | 2 | 2 | | |
| MAPK1 ---> FURIN | | Expression | However, in the presence of T3, furin activation is modulated by MEK/ERK., ... transforming growth factor-ß1 and is regulated by it.4 Even though proprotein convertase5 and PC7 have several structural, biochemical, and cell biological similarities to furin, transforming growth factor-ß1 did not increase the levels of proprotein convertase5 | info:pmid/18467449#body:414, info:pmid/11882580 | 2 | 2 | | |

| | | | | | | | | |
|---|---|---|---|---|---|---|---|---|
| | | | or PC7 mRNAs.4 In vascular endothelial cells, furin increases with increased fluid shear stress, whereas proprotein convertase5 remains unaffected.25 Furthermore, studies in other cell lines report ERK-mitogen-activated protein kinase ... | #body:163 | | | | |
| FURIN ---> REN | | Prot Modification | In contrast, the nanobodies could inhibit only the furin-mediated processing of renin and not that of other PCs., As shown in Fig. 1, renin cleaved from the prorenin-furin precursor was predominant in the 2-h labeling supernatants, suggesting that prosegment cleavage occurred in the constitutive secretory pathway. | info:pmid/22920187#cont:188, info:pmid/8702811#body:129 | 2 | 2 | | |
| ACE2 ---> inflammatory response | | Regulation | We hypothesized that ACE2 overexpression may inhibit inflammation response in atherosclerotic plaque by degrading Ang II into Ang-(1-7)., In atherosclerosis-prone apolipoprotein E knockout mice, ACE2 deficiency results in | info:pmid/19961735#abs:2, info:pmid | 2 | 2 | | |

| | | | | | | | | |
|---|---|---|---|---|---|---|---|---|
| | | | augmented vascular inflammation and an inflammatory response that contributes to increased atherosclerotic plaque formation. | /21099686#abs:4 | | | | |
| ACE2 ---\| SLC2A4 | | Expression | The expression of GLUT4 and MEF2A was increased by angiotensin 1-7 in ACE2 knockout mice and decreased by A779 in wild-type mice., (1) demonstrated that regulation of myocyte enhancer factor 2A and GLUT4 expression by the ACE type 2/angiotensin 1–7/Mas receptor axis contribute to the improvement of insulin sensitivity. | info:pmid/22933108#abs:7, info:pmid/23801722#cont:13 | 2 | 2 | | |
| HIF1A ---\| ACE2 | Pulmonary artery {Organ urn:agi-ncimorgan:C0034052}, Brain {Organ urn:agi-ncimorgan:C1269537} | Regulation | Thus HIF-1alpha inhibited ACE2 expression, and the accumulated ANG II catalyzed by ACE is a key mediator in the downregulation of ACE2 by HIF-1alpha., One potential mechanism may involve transcriptional repression of ACE2 by hypoxia-inducible factor 1a or activation by hepatocyte nuclear factor 1ß (29,30). | info:pmid/19592460#abs:9, info:pmid/22289845#cont:395 | 2 | 2 | | |

| | | | | | | | | |
|---|---|---|---|---|---|---|---|---|
| ACE2 --+> CDH1 | Kidney {Organ urn:agi-ncimorgan:C1278978} | Expression | This is the first study to elucidate the mechanism through which the overexpression of ACE2 in the A549 lung cancer cell line decreases metastasis formation in vivo and upregulates the expression of E-cadherin both in vitro and in vivo., angiotensin II–mediated renal fibrosis was also associated with decreased membrane-fractionated E-cadherin protein levels consistent with epithelial-to-mesenchymal transition, which was prevented by recombinant human ACE2 (Supplemental Figure SIV, available online at http://hyper. ahajournals.org). | info:pmid/23545945#abs:6, info:pmid/21189404#cont:447 | 2 | 2 | | |
| ACE2 --+> viral reproduction | | Regulation | Together, these observations suggest that the affinity of S protein for ACE2 is an important determinant in the overall rate of viral replication and in the severity of disease., Replication of the virus in cells and the formation of syncitia can be blocked by antibodies against ACE2, which indicates the importance of ACE2 in the replication of Severe | info:pmid/15791205#body:246, info:pmid/15464852 | 2 | 3 | | |

| | | | | | | | | |
|---|---|---|---|---|---|---|---|---|
| | | | Acute Respiratory Syndrome-CoV ( )., Importantly, syncytia formation/membrane fusion and viral replication can be specifically inhibited by an anti-angiotensin-converting enzyme-2 antibody ( ) or a fragment containing the receptor binding domain ( ) or antibodies recognizing the receptor binding domain ( ). | #body:99, info:pmid/15708633#body:39 | | | | |
| FURIN ---> MMP2 | | Prot Modification | Furin-cleaved MMP-2 does not possess proteolytic activity as examined in a cell-free assay., Furin can activate matrix metalloproteinases and cleave pro-MMP-2 or -9 to MMP-2 or -9 (37, 38)., of MMP-2 was used as a positive control for furin activity, as MMP-2 is activated by furin target MMP-14, and is cleaved by furin as well.27 | info:pmid/15637056#abs:5, info:pmid/18467449#body:298, info:pmid/22330140#co | 2 | 3 | | |

| | | | | | | | | |
|---|---|---|---|---|---|---|---|---|
| | | | | nt:266 | | | | |
| FURIN ---\| NPPB | | Expression | If furin converts ?BNP to BNP-45, a higher level of furin expression may result in the increased production of BNP-45., Furin and corin seem to be involved in the degradation of proBNP into BNP 1-32 and NT-proBNP 1-76 within or during secretion from the cardiomyocyte., Prohormone convertases such as furin and corin seem to be involved in the degradation of pro-B-type natriuretic peptide into BNP 1-32 and NT-pro-B-type natriuretic peptide 1-76 within or during secretion from the cardiomyocyte. | info:pmid/9001393#body:123, info:pmid/23470072#cont:64, info:pmid/19147726#body:81 | 2 | 3 | | |

| | | | | | | | | |
|---|---|---|---|---|---|---|---|---|
| FURIN ---> INS | | Expression | Having determined that the cell lines produced insulin in response to glucose, we asked if production of mature insulin in the engineered cells was uniquely dependent on furin activity, which is produced in glucose-regulated manner and ultimately is responsible for production of mature insulin., The furin sites allow a ubiquitous endopeptidase to process proinsulin made in the non-ß-cells, facilitating the secretion of mature insulin, the biologically active form of the peptide, to facilitate the production and secretion of mature insulin in human non-ß-cells. <more data available...> | info:pmid/17920636#body:135, info:pmid/16403445#body:27, info:pmid/20719072#cont:203 | 2 | 3 | | |
| ACE2 ---\| matrix metalloproteinase | Heart {Organ urn:agi-ncimorgan:C1281570}, Aorta {Organ urn:agi-ncimorgan:C1278934} | Expression | More intriguingly, recombinant human ACE2 treatment significantly abolished AngII-mediated increases in MMP2, MMP9 and MT1-matrix metalloproteinase in cardiofibroblasts., Overexpression of ACE2 inhibits invasion and Matrix metalloproteinase | info:pmid/24161906#abs:8, info:pmid/21769 | 2 | 3 | | |

| | | | | | | | | |
|---|---|---|---|---|---|---|---|---|
| | | | production in vitro., Along with this concept, Thomas et al12 now show that ACE2 knockout mice have increased aortic expression of matrix metalloproteinases-2 and matrix metalloproteinases-9, as well as increased matrix metalloproteinases-9 production in macrophages. | 437#cont:160, info:pmid/20884883#cont:47 | | | | |
| FGF23 ---\| ACE2 | Kidney {Organ urn:agi-ncimorgan:C1278978} | Expression | Finally, we found that FGF23 suppresses angiotensin-converting enzyme 2 (ACE2) expression in the kidney, thereby providing a pathway for FGF23 regulation of the renin-angiotensin system., First, there is evidence that FGF23 may regulate the renin angiotensin system via suppression of ACE2 expression in the kidney., In this regard, FGF23 appears to be a potent inhibitor of ACE2 expression in the kidney (11), which prevents degradation of Ang I and Ang II. | info:pmid/22970174#abs:7, info:pmid/23465500#body:162, info:pmid/23298840#cont:30 | 2 | 3 | | |

| | | | | | | | | |
|---|---|---|---|---|---|---|---|---|
| FURIN ---> Virus Diseases | | Regulation | Novel furin inhibitors or modified forms of D6R may promote the reduction of immune tolerance and the elimination of infected hepatocytes in patients with chronic hepatitis B virus infection., Various studies have confirmed that furin plays a crucial role in many bacterial and viral diseases, tumorigenesis, neurodegenerative disorders and diabetes., Therefore, furin inhibitors hold great promise as potential therapeutic agents for treating furin-mediated diseases and viral and bacterial infections, particularly for short-term therapy. <more data available...> | info:pmid/23617302#abs:10, info:pmid/21168329#body:3, info:pmid/17179036#body:53, info:pmid/12832286#body:85 | 2 | 4 | | Neighbors of Virus Diseases |

| | | | | | | | | | |
|---|---|---|---|---|---|---|---|---|---|
| TMPRSS2 --+> cell fusion | | Regulation | Directed protease expression and inhibition analyses revealed that TMPRSS2 and endosomal cathepsins activate EMC-S for virus-cell fusion and constitute potential targets for antiviral intervention., Indeed, engineered expression of TMPRSS2 and HAT rendered 229E S-protein-driven virus-cell fusion insensitive to an inhibitor of cathepsin L, a protease previously shown to facilitate human coronavirus 229E infection., However, TMPRSS2 but not human airway trypsin-like protease expression rendered SARS-S-driven virus-cell fusion independent of cathepsin activity, indicating that human airway trypsin-like protease and TMPRSS2 activate SARS-S differentially. <more data available...> | info:pmid/23468491#abs:6, info:pmid/23536651#abs:5, info:pmid/21994442#abs:7, info:pmid/24121034#body:113 | 2 | 4 | | covid |

| | | | | | | | | |
|---|---|---|---|---|---|---|---|---|
| TMPRSS2 --+> viral entry | | Regulation | Thus, TMPRSS2 affects the entry of virus but not other phases of virus replication., Simultaneous treatment with inhibitors of cathepsin L and TMPRSS2 completely blocked virus entry into Vero-TMPRSS2 cells, indicating that Middle East respiratory syndrome coronavirus employs both the cell surface and the endosomal pathway to infect Vero-TMPRSS2 cells., Gierer et al. used VSV-luciferase pseudotyped with the Middle East respiratory syndrome coronavirus S to determine that Middle East respiratory syndrome coronavirus does not utilize any other coronaviral receptors, that TMPRSS2 and endosomal cathepsins facilitate viral entry, and that the S protein can be neutralized with Middle East respiratory syndrome coronavirus infected patient serum ( ). <more data available...> | info:pmid/20926566#abs:6, info:pmid/24027332#abs:6, info:pmid/24269477#body:157, info:pmid/24473083#cont:461 | 2 | 4 | | |

| | | | | | | | | |
|---|---|---|---|---|---|---|---|---|
| TMPRSS2 ---> Infection | Lung {Organ urn:agi-ncimorgan:C1278908} | Regulation | These observations suggest camostat as a candidate antiviral drug to prevent or depress TMPRSS2-dependent infection by severe acute respiratory syndrome coronavirus., Accordingly, coexpression of the 1918 hemagglutinin with TMPRSS4 or the previously identified hemagglutinin-processing protease TMPRSS2 allowed trypsin-independent infection by pseudotypes bearing the 1918 hemagglutinin, indicating that these proteases might support 1918 influenza virus spread in the lung., In a recent study, it was described that the inhibition of TMPRSS2 by the non-specific serine protease inhibitor camostat caused a 10-fold reduction in infection of Calu-3 cells by SARS-CoV [19]. <more data available...> | info:pmid/22496216#abs:6, info:pmid/19158246#abs:8, info:pmid/23527573#cont:51, info:pmid/24121034#body:167 | 2 | 4 | | covid |
| FURIN --+> cell fusion | | Regulation | Furin activity is required for BeWo cell fusion in vitro., A) Inhibition of syncytin 1- and syncytin 2-mediated cell fusion by furin inhibitor I., Finally, we showed that | info:pmid/23598405#co | 2 | 4 | | |

| | | | | | | | | | |
|---|---|---|---|---|---|---|---|---|---|
| | | | CREB mediated furin activation was critical during trophoblast cell fusion process. | nt:43, info:pmid/18650494#body:196, info:embase/2013417921#cont:43, info:doi/10.1016/j.placenta.2012.06.016#body:3663 | | | | | |

| | | | | | | | | |
|---|---|---|---|---|---|---|---|---|
| ACE2 ---> MMP2 | Heart {Organ urn:agi-ncimorgan:C1281570}, Aorta {Organ urn:agi-ncimorgan:C1278934} | Expression | The specific mechanisms by which ACE2 regulates MMP-2, however, remain unclear., More intriguingly, recombinant human ACE2 treatment significantly abolished AngII-mediated increases in MMP2, MMP9 and MT1-matrix metalloproteinase in cardiofibroblasts., MMP-2 activity ( B1 and B2) and MMP2 protein expression level ( C1 and C2) were significantly increased in the Ad-ACE2 group in comparison with the Ad-EGFP and mock groups., Deficiency in ACE2 increases the aortic expression of pro-inflammatory mediators including TNF-a, interleukin-6 (IL-6), MCP-1, vascular cell adhesion molecule 1 (VCAM-1), MMP-2 and MMP-9 , which is accompanied by an increase in adhesion of leucocytes to ECs in vitro and to blood vessel ex vivo . | info:pmid/24219285#abs:3, info:pmid/24161906#abs:8, info:pmid/22340266#body:51, info:pmid/22947420#body:82 | 2 | 4 | | |

| | | | | | | | | |
|---|---|---|---|---|---|---|---|---|
| ACE2 ---> Virus Diseases | | Regulation | Peptides representing various regions of ACE2 critical for virus infection were chemically synthesized and evaluated for antiviral activity., Although the idea is less clear and somewhat controversial, Severe acute respiratory syndrome-coronavirus is thought to use C-type lectins DC-SIGN and/or L-SIGN (collectively referred to as DC/L-SIGN) as alternative receptors or as enhancer factors that facilitate ACE2-mediated virus infection., In addition, siRNAs of TACE and ACE2 blocked viral infection., We then investigated whether a known ACE2-specific peptide inhibitor competed against ACE2-mediated pseudotype virus infection. <more data available...> | info:pmid/16510163#abs:5, info:pmid/17715238#abs:3, info:pmid/18490652#body:47, info:pmid/16033974#body:153, info:pmid/18801550 | 2 | 5 | | |

| | | | | | | | | |
|---|---|---|---|---|---|---|---|---|
| | | | | #body:72 | | | | |
| TMPRSS2 --+> Severe Acute Respiratory Syndrome | | Regulation | In summary, we show that TMPRSS2 might promote viral spread and pathogenesis by diminishing viral recognition by neutralizing antibodies and by activating SARS S for cell-cell and virus-cell fusion., The type II transmembrane protease TMPRSS2 activates the spike (S) protein of severe acute respiratory syndrome coronavirus on the cell surface following receptor binding during viral entry into cells., However, TMPRSS2 but not human airway trypsin-like protease expression rendered SARS-S-driven virus-cell fusion independent of cathepsin activity, indicating that human airway trypsin-like protease and TMPRSS2 activate SARS-S | info:pmid/21325420#abs:7, info:pmid/22496216#abs:1, info:pmid/21994442#abs:7, info:pmid/22222211 | 2 | 5 | | covid |

| | | | | | | | | |
|---|---|---|---|---|---|---|---|---|
| | | | differentially. <more data available...> | #body:81, info:pmid/23527573#cont:49 | | | | |
| ACE2 ---\| vascularization | Aorta {Organ urn:agi-ncimorgan:C1278934} | Regulation | These results suggest that the overexpression of ACE2 may potentially suppress the invasion and angiogenesis of non-small cell lung cancer., Here, we show, both in vitro and in vivo, that ACE2 inhibited the development of early atherosclerotic lesions by suppressing the growth of vascular smooth muscle cells and improving endothelial function., A recent study showed that ACE2 could inhibit the angiogenesis of non-small cell lung cancer by decreasing VEGF ., Similarly, recent evidence suggests that the overexpression of ACE2 may influence angiogenesis by inhibiting inflammation, cell growth, and VEGFa production in vitro . <more data available...> | info:pmid/21769437#abs:8, info:pmid/20798044#abs:3, info:pmid/21481527#body:125, info:pmid | 2 | 5 | | |

| | | | | | | | | | |
|---|---|---|---|---|---|---|---|---|---|
| | | | | /22749485#body:78, info:pmid/22947420#body:68 | | | | | |
| FURIN ---> FGF23 | Kidney {Organ urn:agi-ncimorgan:C1278978} | Prot Modification | By this model, furin-mediated FGF23 processing would be increased in the iron-deficient state to guard against hypophosphatemia., Furin, a subtilisin-like proprotein convertase enzyme, cleaves intact FGF-23 and generates smaller N-terminal (18 kDa) and C-terminal (12 kDa) fragments (27)., The site of incorporation was determined by MALDI-TOF analysis of the furin-cleaved product of the FGF23b GalNAc glycopeptide, where the N-terminal peptide fragment was found as the expected mass (calculated mass 1032.2) plus the saccharide component | info:pmid/22921867#body:122, info:pmid/22573526#cont:245, info:pmid/16638743 | 2 | 5 | | |

| | | | | | | | | |
|---|---|---|---|---|---|---|---|---|
| | | | (calculated mass 1235) (Fig. 3). <more data available...> | #body:161, info:pmid/12506157#body:219, info:pmid/20837471#cont:238 | | | | |
| ACE2 ---\| MMP9 | Heart {Organ urn:agi-ncimorgan:C1281570}, Aorta {Organ urn:agi-ncimorgan:C1278934} | Expression | More intriguingly, recombinant human ACE2 treatment significantly abolished AngII-mediated increases in MMP2, MMP9 and MT1-matrix metalloproteinase in cardiofibroblasts., We also found that the overexpression of ACE2 suppressed A549 cell invasion and MMP-2 and MMP-9 activity in vitro and in vivo., In our study, genetic Ace2 deficiency was associated with | info:pmid/24161906#abs:8, info:pmid/21769437#cont:217, | 2 | 6 | | |

| | | | | | | | | | |
|---|---|---|---|---|---|---|---|---|---|
| | | | increased aortic expression of gelati-nases, matrix metalloproteinase-2 (gelatinase-A) and MMP-9 (gelatinase-B), and augmented production of MMP-9 in macrophages. <more data available...> | info:pmid/20671240#cont:234, info:pmid/20798044#cont:215, info:pmid/22947420#body:82, info:pmid/18403726#body:195 | | | | | |

| | | | | | | | | |
|---|---|---|---|---|---|---|---|---|
| EDN1 ---\| ACE2 | Bronchi {Organ urn:agi-ncimorgan:C0006255}, Heart {Organ urn:agi-ncimorgan:C1281570} | Expression | Endothelin-1 (ET-1) also significantly reduced myocyte ACE2 mRNA., ET-1 downregulates ACE2 expression and activity at the transcription level in human bronchial epithelial cells via the endothelin A receptor by a p38 mitogen-activated protein kinase-dependent mechanism., This finding supports results of a previous study whereby ET-1 treatment at 10 nM for 12 h reduced ACE2 mRNA by ~60% in cardiomyocytes (21)., In cardiac myocytes and fibroblasts, ACE-2 mRNA and protein are downregulated by ANGII or endothelin -1 and upregulated by the angiotensin receptor AT1 blocker losartan [26]. <more data available...> | info:pmid/18849338#abs:6, info:pmid/23751363#abs:6, info:pmid/23836146#cont:190, info:pmid/23100504#cont:306, info:pmid/21045 | 2 | 6 | | |

| | | | | | | | | |
|---|---|---|---|---|---|---|---|---|
| | | | | 683#cont:64, info:pmid/21881224#cont:228 | | | | |
| ACE2 ---> heart development | Heart {Organ urn:agi-ncimorgan:C1281570}, Epicardium {Organ urn:agi-ncimorgan:C0225968}, Blood Vessels {Organ urn:agi-ncimorgan:C0005847}, Coronary artery {Organ urn:agi-ncimorgan:C1269008} | Regulation | Perspectives The present study demonstrated that ACE2 might suppress the development of cardiac hypertrophy and congestive heart failure induced by pressure overload., In support of this possibility is evidence that deletion of the ACE2 gene leads to the development of heart failure and that this effect can be inhibited by further deletion of the ACE gene ( )., A previous study suggested that ACE2 is involved in cardiac development, whereby the ACE2 promoter is activated in epicardial cells in Xenopus embryos via a GATA-binding site (39). <more data available...> | info:pmid/16505206#body:233, info:pmid/18718424#body:25, info:pmid/24177423#cont:154 | 2 | 7 | | |

| | | | | | | | | |
|---|---|---|---|---|---|---|---|---|
| | | | | , info:pmid/22523556#cont:186, info:pmid/23249272#cont:183, info:pmid/23608725#body:94, info:doi/10.1016/j.cardfail.2007.06. | | | | |

| | | | | | | | | |
|---|---|---|---|---|---|---|---|---|
| | | | | 303#body:1 | | | | |
| FURIN ---> INS | Liver {Organ urn:agi-ncimorgan:CL384198}, Pancreas {Organ urn:agi-ncimorgan:C1278931} | Prot Modification | Furin protease cleaved insulin peptides in vitro., Since the biochemical characteristics of the insulin proreceptor processing endopeptidase activity mostly resembled those of furin activity, it is likely that insulin proreceptor proteolytic maturation can be catalysed by furin in the liver., In these experiments a tetracycline-inducible promoter was inserted above the furin-modified insulin coding sequence., Proinsulin cleaved by furin is processed to chromatographically mature insulin by carboxypeptidases in nonneuroendocrine cells. <more data available...> | info:pmid/21143365#abs:10, info:pmid/8037679#abs:11, info:pmid/11593361#body:184, info:pmid/88447 | 2 | 7 | | |

| | | | | | | | | | |
|---|---|---|---|---|---|---|---|---|---|
| | | | | 68#titl e:1, info:pmid/12085245#body:59, info:pmid/17920636#body:104, info:pmid/8995442#body:48 | | | | | |
| FURIN ---> NPPB | Kidney {Organ urn:agi-ncimorgan:C1278978}, Heart {Organ urn:agi-ncimorgan:C1281570}, Heart Atrium {Organ urn:agi-ncimorgan:C0018792} | Prot Modification | Previous studies showed that both furin and corin cleaved pro-BNP., There is evidence to suggest that amphibian BNP precursors are probably cleaved by the endoprotease furin, which recognises a specific amino acid | info:pmid/21763278#body:64, inf | | 2 | 8 | | |

| | | | | | | | | | |
|---|---|---|---|---|---|---|---|---|---|
| | | | sequence Arg-X-X-Arg ( )., In this pathway, the BNP precursor is thought to be cleaved by furin, because the BNP precursor possesses a furin-cleavable RXXR sequence at its processing site (19)., Pro-BNP is cleaved by corin or furin, mainly in the cytoplasm of cardiac myocytes, to yield to N-terminal (NT-proBNP) and C-terminal (BNP) portions of proBNP. <more data available...> | o:pmid/16343494#body:25, info:pmid/9252368#body:51, info:pmid/9252369#body:51, info:pmid/23684562#body:7, info:pmid/15265821 | | | | | |

| | | | | | | | | |
|---|---|---|---|---|---|---|---|---|
| | | | | #body:146, info:pmid/19147726#body:54, info:doi/10.1016/j.cardfail.2011.06.076#body:1 | | | | |
| ACE2 ---\| MAPK1 | umbilical artery {Organ urn:agi-ncimorgan:C0041632}, Left ventricular {Organ urn:agi-ncimorgan:C0225897}, Aorta {Organ urn:agi-ncimorgan:C1278934}, Heart {Organ urn:agi-ncimorgan:C1281570}, Kidney {Organ urn:agi-ncimorgan:C1278978} | Regulation | More importantly, treatment with human recombinant ACE2 (1mg/ml) dramatically prevented Angiotensin II-mediated SOCS3 expression and the JAK2-STAT3 and ERK1/2 signaling, and resulted in attenuation of superoxide production and cell proliferation in Human umbilical artery | info:pmid/23816468#abs:7, info:pmid/20854 | 2 | 8 | | |

| | | | | | | | | | |
|---|---|---|---|---|---|---|---|---|---|
| | | | smooth muscle cells., Importantly, treatment with telmisartan (1 or 10 µM) or recombinant human ACE2 (2mg/ml) largely ameliorated angiotensin II-induced profilin-1 expression and extracellular-signal regulated kinase 1/2 and JNK phosphorylation and augmented PPAR? ?expression in the cultured human umbilical artery smooth muscle cells. <more data available...> | 846#abs:7, info:pmid/22595130#body:108, info:pmid/22693641#cont:139, info:pmid/20679547#body:150, info:pmid/24161906#cont: | | | | |

| | | | | | | | | |
|---|---|---|---|---|---|---|---|---|
| | | | | 195, info:pmid/21189404#cont:437, info:doi/10.1016/j.peptides.2012.01.020#body:16 | | | | |
| ACE2 ---\| TGFB1 | Kidney {Organ urn:agi-ncimorgan:C1278978}, Cardiovascular system {Organ urn:agi-ncimorgan:C1269562}, Islets of Langerhans {Organ urn:agi-ncimorgan:C0022131} | Expression | After 8weeks of treatment, compared with Goldblatt group, felodipine+puerarin reduced SBP, DBP and HR (p<0.01 or p<0.05), ameliorated renal interstitial fibrosis, decreased the level of Ang II and increased that of Ang (1-7), upregulated mRNA expression of ACE2 and | info:pmid/23523569#abs:5, info:pmid/20679 | 2 | 8 | | |

| | | | | | | | | |
|---|---|---|---|---|---|---|---|---|
| | | | Mas, decreased that of ACE and AT1, and downregulated protein expression of TGF-ß1 in kidneys (p<0.01)., Ang II–mediated myocardial fibrosis and expression of procollagen type Ia1, procollagen type IIIa1, transforming growth factor-ß1, and fibronectin were also suppressed by recombinant human ACE2. <more data available...> | 547#cont:19, info:pmid/23174757#body:18, info:pmid/22340267#body:29, info:pmid/21189404#cont:635, info:pmid/22340266#body:66, | | | | |

| | | | | | | | | |
|---|---|---|---|---|---|---|---|---|
| | | | | info:pmid/18948167#body:114, info:doi/10.1016/j.cardfail.2010.06.054#body:5 | | | | |
| FURIN --+> cell invasion | Veins {Organ urn:agi-ncimorgan:C0042449}, Liver {Organ urn:agi-ncimorgan:CL384198}, Endometrium {Organ urn:agi-ncimorgan:C1550633} | Regulation | Furin complementation resulted in an increased cell invasiveness that correlated with their capacity to produce MMP-2., Our results demonstrated that the furin inhibitor decreased pro-MT1-MMP processing as well as pro-MMP-2 activation and cell invasiveness., In contrast, over-expression of furin markedly increased cell invasion and migration | info:pmid/14644155#abs:6, info:pmid/9539163#abs:6, | 2 | 10 | | Neighbors of cell invasion |

| | | | | | | | | | |
|---|---|---|---|---|---|---|---|---|---|
| | | | (P<0.01), accompanied by significant increase of MMP-9 activities., Furin over-expression could occur in liver cancer and a previous study showed that over-expression of furin promoted HepG2 cell invasion in tail vein xenograft models. <more data available...> | info:pmid/19853298#abs:7, info:pmid/22808247#abs:3, info:pmid/23835774#cont:206, info:pmid/18467449#body:349, info:pmid/16194 | | | | | |

| | | | | | | | | |
|---|---|---|---|---|---|---|---|---|
| | | | | 539#body:124, info:pmid/23598405#cont:27, info:doi/10.1016/S0014-4827(03)00407-5#body:169, info:embase/2013417921#con | | | | |

| | | | | | | | | | |
|---|---|---|---|---|---|---|---|---|---|
| | | | | t:27 | | | | | |
| FURIN ---> EDN1 | Lower jaw region {Organ urn:agi-ncimorgan:C0460026}, Bone and Bones {Organ urn:agi-ncimorgan:C0262950}, female reproductive system {Organ urn:agi-ncimorgan:C0700038}, Heart {Organ urn:agi-ncimorgan:C1281570} | Prot Modification | Analysis of the processing of precursors of endothelin-1 (ET-1), adrenomedullin , transforming growth factor ß1 (TGF-ß1), and bone morphogenetic protein 4 (BMP4) confirmed that ET-1, adrenomedullin, and TGF-ß1 are in vivo substrates of endothelial furin., These studies suggest that Edn1 is cleaved twice, first by Furin and second by ECE1 and that these cleavages are required for Edn1 bioactivity., Bone morphogenic protein 7 and endothelin-1 were both glycosylated and cleaved by furin in vitro but glycosylation did not yield protection against processing. <more data available...> | info:pmid/22733989#abs:9, info:pmid/17574232#body:17, info:pmid/21937429#cont:293, info:pmid | 2 | 10 | | | |

| | | | | | | | | |
|---|---|---|---|---|---|---|---|---|
| | | | | /22553222#cont:82, info:pmid/22406696#body:17, info:pmid/16678149#body:18, info:pmid/23470073#cont:21, info:pmid/11577023#body: | | | | |

| | | | | | | | | |
|---|---|---|---|---|---|---|---|---|
| | | | | 72, info:pmid/11067800#body:69, info:doi/10.1016/j.drudis.2012.02.017#body:17 | | | | |
| ACE2 ---> APLN | Cardiovascular system {Organ urn:agi-ncimorgan:C1269562}, hypothalamus {Organ urn:agi-ncimorgan:C0020663}, Heart {Organ urn:agi-ncimorgan:C1281570} | Prot Modification | Apelin is a second catalytic substrate for ACE2 and functions as an inotropic and cardioprotective peptide., Apelin is also a catalytic substrate for angiotensin-converting enzyme 2, the key severe acute respiratory syndrome receptor., ACE2 also cleaves some peptides, such as dynorphin, apelin and bradykinin., ACE2 most efficiently cleaves | info:pmid/24177423#abs:2, info:pmid/17673668#abs:2, | 2 | 12 | | |

| | | | | | | | | |
|---|---|---|---|---|---|---|---|---|
| | | | apelin-13, dynorphin A (1-13), and des-Arg9 bradykinin (4, 5)., Specific cleaving of apelin peptides by ACE2, the first human homologue of angiotensin-converting enzyme (ACE), has been observed ( ). <more data available...> | info:pmid/20700837#cont:78, info:pmid/16166094#body:70, info:pmid/15907343#body:185, info:pmid/11815627#body:220, info:pmid | | | | |

| | | | | | | | | |
|---|---|---|---|---|---|---|---|---|
| | | | | /20605969#cont:73, info:pmid/23962453#body:18, info:pmid/16298469#body:8, info:pmid/21099686#cont:56 <more data available...> | | | | |

| | | | | | | | | |
|---|---|---|---|---|---|---|---|---|
| FURIN ---\| cell differentiation | Liver {Organ urn:agi-ncimorgan:CL384198}, Ectoderm {Organ urn:agi-ncimorgan:C0013574}, Stomach {Organ urn:agi-ncimorgan:C1278920}, Cochlea {Organ urn:agi-ncimorgan:C0009195}, Epidermis {Organ urn:agi-ncimorgan:C0014520} | Regulation | Thus, furin appears to control the proliferation as well as differentiation of islet cells., Thus, we conclude that Interleukin 12 induction of furin might represent a new aspect of IFN-gamma regulation and control of T helper 1 differentiation., The liver produces a number of proproteins having a furin-cleavable site; thus, furin may be involved in growth and differentiation both in the partially hepatectomized liver and in primary cultured hepatocytes., These results indicate that Sty1/Spc1 controls the cellular differentiation process by controlling Atf1. <more data available...> | info:pmid/8895387#abs:12, info:pmid/16627761#abs:6, info:pmid/9426209#abs:2, info:pmid/9585499#body:131, info:pmid/21896659#co | 2 | 13 | | Neighbors of cell differentiation |

| | | | | | | | | | |
|---|---|---|---|---|---|---|---|---|---|
| | | | | nt: 125 , info:pmid/23545100#body:110 , info:pmid/9109428#title:1, info:pmid/18713856#body:237 , info:pmid/23277081#cont:88, inf | | | | | |

| | | | | | | | | |
|---|---|---|---|---|---|---|---|---|
| | | | | o:pmid/15505202#body:137 <more data available...> | | | | |
| FURIN ---> matrix metalloproteinase | Kidney Glomerulus {Organ urn:agi-ncimorgan:C0022663} | Prot Modification | These data indicate that furin can directly cleave Matrix metalloproteinases containing an RXXR motif., Furthermore, the matrix metalloprotease MMP-14, which is PMA-inducible (reviewed in Ref. 34), is cleaved by furin., Indeed, furin was demonstrated to cleave this sequence in prostromelysin-3 and proMT1-Matrix metalloproteinase resulting in enzyme activation ., The presence of an RXK/RR motif suggests that the prodomain of CA-matrix metalloproteinase will be cleaved off by furin or related enzymes in | info:pmid/15637056#body:244, info:pmid/19047044#body:307, info:pmid/90371 | 2 | 13 | | |

| | | | | | | | | |
|---|---|---|---|---|---|---|---|---|
| | | | the trans-Golgi network. <more data available...> | 99#bo dy: 5, info:pmid/10471791#body:150, info:pmid/19166965#body:55, info:pmid/15560752#body:243, info:pmid/17332756#body: | | | | |

| | | | | | | | | |
|---|---|---|---|---|---|---|---|---|
| | | | | 52, info:pmid/12665557#body:299, info:pmid/20686912#cont:132, info:pmid/20605060#body:122<more data available...> | | | | |

| | | | | | | | | |
|---|---|---|---|---|---|---|---|---|
| ACE2 ---> Infection | Heart {Organ urn:agi-ncimorgan:C1281570}, Lung {Organ urn:agi-ncimorgan:C1278908} | Regulation | The soluble ectodomain of ACE2 specifically abrogated S-mediated infection and might therefore be exploited for the generation of inhibitors., Finally, we show that a soluble and catalytically inactive form of ACE2 potently blocked infection by S-protein-pseudotyped retrovirus and by severe acute respiratory syndrome-CoV., Pulmonary infection with the human severe acute respiratory syndrome-CoV in mice led to an ACE2-dependent myocardial infection with a marked decrease in ACE2 expression confirming a critical role of ACE2 in mediating severe acute respiratory syndrome-CoV infection in the heart. <more data available...> | info:pmid/15194496#abs:3, info:pmid/15367630#abs:7, info:pmid/19453650#abs:5, info:pmid/16730806#body:9, info:pmid/16337697#body: | 2 | 16 | | |

| | | | | | | | | |
|---|---|---|---|---|---|---|---|---|
| | | | | 13, info:pmid/16510163#body:57, info:pmid/15897467#body:115, info:pmid/23962453#body:64, info:pmid/16033974#body:129, info:p | | | | |

| | | | | | | | | |
|---|---|---|---|---|---|---|---|---|
| | | | | mid/15381196#body:65 <more data available...> | | | | |
| FURIN ---> TGFB1 | Salivary Glands {Organ urn:agi-ncimorgan:C0036098}, Bone and Bones {Organ urn:agi-ncimorgan:C0262950}, Respiratory System {Organ urn:agi-ncimorgan:C1269561}, Prostate {Organ urn:agi-ncimorgan:C1278980}, Brain {Organ urn:agi-ncimorgan:C1269537} | Prot Modification | Analysis of the processing of precursors of endothelin-1 (ET-1), adrenomedullin , transforming growth factor ß1 (TGF-ß1), and bone morphogenetic protein 4 (BMP4) confirmed that ET-1, adrenomedullin, and TGF-ß1 are in vivo substrates of endothelial furin., TGF-ß positively regulates expression of furin and is a furin substrate (64) ., As with TGFß ligands, the inhibin precursor molecules are apparent substrates for furin cleavage., Furin has traditionally been suggested to cleave pro-TGF-ß intracellularly during processing in the | info:pmid/22733989#abs:9, info:pmid/12832286#body:150, info:pmid/18826948#body: | 2 | 20 | | |

| | | | | | | | | |
|---|---|---|---|---|---|---|---|---|
| | | | golgi apparatus. <more data available...> | 54, info:pmid/18826955#body:54, info:pmid/18243766#body:41, info:pmid/15975431#body:224, info:pmid/24098777#cont:72, info:pmid | | | | |

| | | | | | | | | |
|---|---|---|---|---|---|---|---|---|
| | | | | /17516499#title:1, info:pmid/22808247#cont:282, info:pmid/20870411#body:14 <more data available...> | | | | |

| | | | | | | | | |
|---|---|---|---|---|---|---|---|---|
| ACE2 ---\| Atherosclerosis | Blood Vessels {Organ urn:agi-ncimorgan:C0005847}, Kidney {Organ urn:agi-ncimorgan:C1278978}, Arteries {Organ urn:agi-ncimorgan:C0003842}, Cerebrum {Organ urn:agi-ncimorgan:C1280654}, Carotid Arteries {Organ urn:agi-ncimorgan:C0007272}, Aorta {Organ urn:agi-ncimorgan:C1278934}, Heart {Organ urn:agi-ncimorgan:C1281570} | Regulation | ACE2 deficiency increased atherosclerotic area (Ace2(+/y), 17 ± 1; Ace2(-/y), 23 ± 2 mm(2), P < 0.002)., However, the underlying mechanisms by which ACE2 effectively suppresses early atherosclerotic lesions remain poorly understood., Angiotensin converting enzyme-2 confers endothelial protection and attenuates atherosclerosis., ACE2 degrades pro-atherosclerotic Ang II and increases anti-atherosclerotic angiotensin 1-7., (2008) Angiotensin converting enzyme-2 confers endothelial protection and attenuates atherosclerosis. <more data available...> | info:pmid/21252069#abs:5, info:pmid/20798044#abs:2, info:pmid/18660448#title:1, info:pmid/22947420#body:141, info:pmid/23249272 | 2 | 23 | | |

| | | | | | | | | |
|---|---|---|---|---|---|---|---|---|
| | | | | #cont:351, info:pmid/24147777#cont:265, info:pmid/22659526#cont:350, info:pmid/20599443#body:60, info:pmid/23266545#cont: | | | | |

| | | | | | | | | |
|---|---|---|---|---|---|---|---|---|
| | | | | 6, info:pmid/24193738#title:1 <more data available...> | | | | |
| ACE2 ---> Severe Acute Respiratory Syndrome | Lung {Organ urn:agi-ncimorgan:C1278908} | Regulation | Importantly, ACE2 has been identified as a key SARS-coronavirus receptor and plays a protective role in SARS pathogenesis., Finally, we show that a soluble and catalytically inactive form of ACE2 potently blocked infection by S-protein-pseudotyped retrovirus and by severe acute respiratory syndrome-CoV., Infection by severe acute respiratory syndrome coronavirus is initiated by specific interactions between the severe acute respiratory syndrome coronavirus spike (S) protein and its receptor ACE2., Monkey and | info:pmid/20134095#abs:4, info:pmid/15367630#abs:7, info:pmid/19853613#abs:1, | 2 | 24 | | |

| | | | | | | | | |
|---|---|---|---|---|---|---|---|---|
| | | | human kidney cells (LLC-MK2, Vero, and 769-P) and swine kidney cells were permissive for both viruses, but only severe acute respiratory syndrome-CoV infection could be blocked by anti-human ACE2 antibody and could be neutralized by preincubation of virus with soluble ACE2. <more data available...> | info:pmid/23232719#abs:8, info:pmid/18554741#body:172, info:pmid/23962453#body:64, info:pmid/15980414#body:65, info:pmid/18801 | | | | |

| | | | | | | | | |
|---|---|---|---|---|---|---|---|---|
| | | | | 550#body:119, info:pmid/21533129#cont:135, info:pmid/24219285#cont:280<more data available...> | | | | |

| | | | | | | | | |
|---|---|---|---|---|---|---|---|---|
| TGFB1 --+> FURIN | Aorta {Organ urn:agi-ncimorgan:C1278934}, Synovial Membrane {Organ urn:agi-ncimorgan:C0039099}, Lung {Organ urn:agi-ncimorgan:C1278908}, Heart {Organ urn:agi-ncimorgan:C1281570}, Arteries {Organ urn:agi-ncimorgan:C0003842}, Pulmonary artery {Organ urn:agi-ncimorgan:C0034052}, Cornea {Organ urn:agi-ncimorgan:C1550625}, artery wall {Organ urn:agi-organ:artery%20wall}, Bone and Bones {Organ urn:agi-ncimorgan:C0262950}, Pituitary Gland {Organ urn:agi-ncimorgan:C1278880} <more data available...> | Expression | Furthermore, TGF-beta itself increased the furin mRNA levels., Although the furin mRNA level was increased by TGF-beta1 in Dami cells, it was not affected by PDGF-BB., Stimulation with TGFbeta1 results in a significant increase in furin mRNA levels, starting at 3 h with the peak effect observed at 12 h (2.5-fold increase +/-0.4)., In primary culture of rat hepatocytes, furin expression increases gradually with time, and its expression is greatly enhanced by transforming growth factor beta1, whose processing from the precursor requires cleavage by furin. <more data available...> | info:pmid/11348875#abs:6, info:pmid/12097169#abs:9, info:pmid/9109442#abs:4, info:pmid/9426209#abs:4, info:pmid/21112328#abs:10, | 2 | 28 | | |

| | | | | | | | | |
|---|---|---|---|---|---|---|---|---|
| | | | | info:pmid/18467449#body:423, info:pmid/22808247#cont:286, info:pmid/12832286#body:150, info:pmid/12177061#body:415, inf | | | | |

| | | | | | | | | |
|---|---|---|---|---|---|---|---|---|
| | | | | o:pmid/17948127#body:106<more data available...> | | | | |
| ACE2 ---\| Hypertension | Kidney {Organ urn:agi-ncimorgan:C1278978}, Brain {Organ urn:agi-ncimorgan:C1269537}, Kidney Glomerulus {Organ urn:agi-ncimorgan:C0022663}, hypothalamic paraventricular nucleus {Organ urn:agi-organ:hypothalamic%20paraventricular%20nucleus}, Medulla Oblongata {Organ urn:agi-ncimorgan:C1269575}, Blood Vessels {Organ urn:agi-ncimorgan:C0005847}, Cardiovascular system {Organ urn:agi-ncimorgan:C1269562}, Heart Ventricle {Organ urn:agi- | Regulation | ACE2 amplification may have a potential therapeutic role for kidney disease and hypertension., This prevention of hypertension by ACE2 overexpression was reversed by blockade of the Ang-(1-7) receptor (d-Ala7-Ang-[1-7]; 600 ng/kg per minute)., Amplifying angiotensin-converting enzyme 2 activity may have a potential therapeutic role for kidney disease and hypertension., In addition, overexpression of ACE2 in the brain reduces hypertension by improving arterial baroreflex and autonomic function., We hypothesized that | info:pmid/18775121#abs:4, info:pmid/19926873#abs:6, info:pmid/18408475#abs:8, inf | 2 | 62 | | |

| | | | | | | | | |
|---|---|---|---|---|---|---|---|---|
| | ncimorgan:C0018827}, Heart {Organ urn:agi-ncimorgan:C1281570}, Coronary artery {Organ urn:agi-ncimorgan:C1269008} <more data available...> | | ADAM17-mediated ACE2 shedding results in decreased membrane-bound ACE2 in the brain, thus promoting the development of neurogenic hypertension. <more data available...> | o:pmid/19124678#abs:10, info:pmid/24014829#abs:3, info:pmid/21952934#abs:4, info:pmid/17325232#abs:11, info:pmid/16878172#abs:9, | | | | |

| | | | | | | | | |
|---|---|---|---|---|---|---|---|---|
| | | | | info:pmid/23968354#abs:9, info:pmid/18022600#abs:1 <more data available...> | | | | |
| ACE2 ---\| renin-angiotensin system | Kidney {Organ urn:agi-ncimorgan:C1278978}, Heart {Organ urn:agi-ncimorgan:C1281570}, Cardiovascular system {Organ urn:agi-ncimorgan:C1269562}, Brain {Organ urn:agi-ncimorgan:C1269537}, Pancreas {Organ urn:agi-ncimorgan:C1278931}, Lung {Organ urn:agi-ncimorgan:C1278908}, Intestines {Organ urn:agi-ncimorgan:C0021853}, | Regulation | It is a consequence of this action that ACE2 participates in the renin-angiotensin system., Angiotensin-converting enzyme 2 (ACE2) is a new component of the renin-angiotensin system ., Angiotensin-converting enzyme-2 (ACE2) is a negative regulator of the renin-angiotensin system., Angiotensin-converting enzyme 2 (ACE2) is a newly identified component of renin- | info:pmid/15549171#abs:4, info:pmid/20424953#abs:1, inf | 2 | 100 | | |

| | | | | | | | |
|---|---|---|---|---|---|---|---|
| | Alveolus {Organ urn:agi-ncimorgan:C1515933}, Vascular system {Organ urn:agi-ncimorgan:C0489903}, Aorta {Organ urn:agi-ncimorgan:C1278934} <more data available...> | | angiotensin system., Angiotensin-converting enzyme 2 (ACE2) is a newly identified regulator of the renin-angiotensin system., Angiotensin-converting enzyme 2 (ACE2) is an important negative regulator of the renin-angiotensin system. <more data available...> | o:pmid/22474255#abs:2, info:pmid/21769437#abs:2, info:pmid/19759332#abs:1, info:pmid/21285291#abs:1, info:pmid/18307733#abs:2, info:p | | | |

| | | | | | | | | |
|---|---|---|---|---|---|---|---|---|
| | | | | mid/17703127#abs:1, info:pmid/23706365#abs:1, info:pmid/22800722#abs:4 <more data available...> | | | | |
